# Extreme Conservation Leads to Recovery of the Virunga Mountain Gorillas

**DOI:** 10.1371/journal.pone.0019788

**Published:** 2011-06-08

**Authors:** Martha M. Robbins, Markye Gray, Katie A. Fawcett, Felicia B. Nutter, Prosper Uwingeli, Innocent Mburanumwe, Edwin Kagoda, Augustin Basabose, Tara S. Stoinski, Mike R. Cranfield, James Byamukama, Lucy H. Spelman, Andrew M. Robbins

**Affiliations:** 1 Department of Primatology, Max Planck Institute for Evolutionary Anthropology, Leipzig, Germany; 2 The International Gorilla Conservation Programme, Kigali, Rwanda; 3 Dian Fossey Gorilla Fund International, Atlanta, Georgia, United States of America; 4 Mountain Gorilla Veterinary Program, School of Veterinary Medicine, University of California Davis, Davis, California, United States of America; 5 Parc National des Volcans, Rwanda Development Board, Gishushu, Kigali, Rwanda; 6 Parc National des Virunga-sud, Institut Congolais pour la Conservation de la Nature, IGCP-DRC, Gisenyi, Rwanda; 7 Mgahinga Gorilla National Park, Uganda Wildlife Authority, Kampala, Uganda; 8 Zoo Atlanta, Atlanta, Georgia, United States of America; University of California, Berkeley, United States of America

## Abstract

As wildlife populations are declining, conservationists are under increasing pressure to measure the effectiveness of different management strategies. Conventional conservation measures such as law enforcement and community development projects are typically designed to minimize negative human influences upon a species and its ecosystem. In contrast, we define “extreme” conservation as efforts targeted to deliberately increase positive human influences, including veterinary care and close monitoring of individual animals. Here we compare the impact of both conservation approaches upon the population growth rate of the critically endangered Virunga mountain gorillas (*Gorilla beringei beringei)*, which increased by 50% since their nadir in 1981, from approximately 250 to nearly 400 gorillas. Using demographic data from 1967–2008, we show an annual decline of 0.7%±0.059% for unhabituated gorillas that received intensive levels of conventional conservation approaches, versus an increase 4.1%±0.088% for habituated gorillas that also received extreme conservation measures. Each group of habituated gorillas is now continuously guarded by a separate team of field staff during daylight hours and receives veterinary treatment for snares, respiratory disease, and other life-threatening conditions. These results suggest that conventional conservation efforts prevented a severe decline of the overall population, but additional extreme measures were needed to achieve positive growth. Demographic stochasticity and socioecological factors had minimal impact on variability in the growth rates. Veterinary interventions could account for up to 40% of the difference in growth rates between habituated versus unhabituated gorillas, with the remaining difference likely arising from greater protection against poachers. Thus, by increasing protection and facilitating veterinary treatment, the daily monitoring of each habituated group contributed to most of the difference in growth rates. Our results argue for wider consideration of extreme measures and offer a startling view of the enormous resources that may be needed to conserve some endangered species.

## Introduction

While the need to show the impact of different conservation strategies is increasingly recognized, such analyses are often difficult or impossible due to a lack of data to assess trends in population dynamics under different conservation regimes and ecological conditions [1]–[5]. In many cases, simply getting an accurate assessment of population sizes may be difficult, even for large, terrestrial megafauna that capture the public’s attention and serve as flagship species for conservation [6], [7]. Conventional conservation measures such as law enforcement and community development projects are typically designed to minimize negative human influences upon a species and its ecosystem. In contrast, we define “extreme” conservation as efforts targeted to deliberately increase positive human influences, including the detection and veterinary treatment of potentially life threatening conditions and close surveillance of individual animals [8], [9]. Assessments of both approaches can be enhanced by understanding the natural and human-induced influences upon the population dynamics of a species. Here we quantify the relative impact of anthropogenic and socioecological influences upon the population growth rate of the Virunga mountain gorillas, a critically endangered primate that has received an extraordinary level of both conservation approaches.

Wild gorilla populations have suffered catastrophic losses in the past two decades, and three of their four subspecies are critically endangered [10], [11]. Mountain gorillas (*Gorilla beringei beringei*) are one of the most critically endangered of all great ape subspecies, with only two isolated populations remaining. One of these populations, the Virunga mountain gorillas, is confined to 450 km^2^ in three contiguous national parks that straddle the borders of Rwanda, Uganda, and the Democratic Republic of Congo. The Virunga Massif is surrounded by some of the highest rural human population densities in the world [12], up to 820 people per km^2^. High human densities can adversely affect local wildlife conservation [13], and the Virunga mountain gorillas have faced multiple threats such as habitat destruction and poaching.

**Figure 1:**
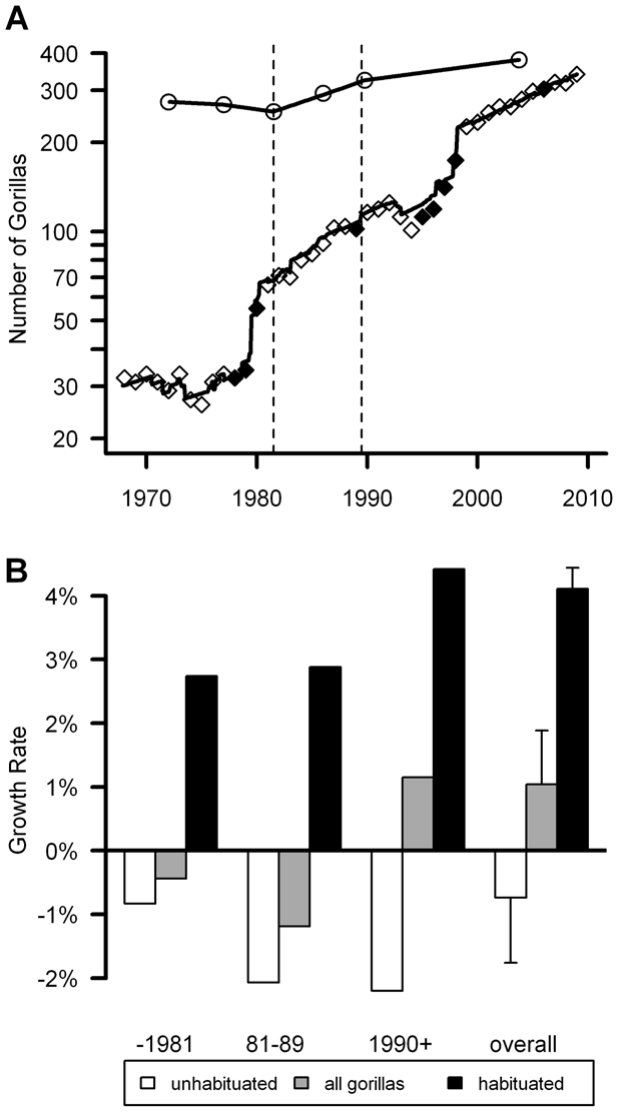
Temporal variations in the population size and growth rate. a. Size of the total population (circles) and the habituated groups (diamonds) throughout the study. Filled diamonds indicate years when additional groups were habituated. Solid lines show the results from the time series analyses for the intervals before the 1972 census, between each pair of consecutive censuses, and after the 2003 census. Dashed vertical lines show the three broader time intervals used in Figure 1b. The overall population density equals the total population size divided by the park area (450 km^2^). b. Time series analyses for the growth rates of the habituated groups, unhabituated groups, and the total population before the 1981 census, after the 1989 census, and during the interval in between. Error bars for the overall growth rates indicate the standard error among the five intervals between consecutive censuses.

This study can be divided into three approximate time periods with different levels of conservation efforts and different threats to the gorillas (dashed vertical lines in Figure 1a). The first of six complete censuses of the Virunga mountain gorillas was conducted in 1971, and the population declined from 275 to 254 gorillas over the next decade due to habitat destruction and poaching (circles in Figure 1a). Mountain gorillas traditionally have not been hunted for bushmeat, but they get caught in snares that poachers set for antelope, and they get killed for other reasons (e.g., when infants are abducted for pets). International focus on the population increased in the late 1970s and 1980s as research findings and conservation challenges were widely publicized. Conservation activities intensified in the 1980s with a multi-pronged approach of local conservation education, law enforcement, an innovative veterinary program, and pioneering efforts to habituate gorillas for ecotourism [14]–[16]. As a result of those efforts, the gorilla population increased to 320 gorillas by 1989. Civil unrest erupted in the 1990s, with armed forces and refugees occupying areas in and around parks to the present [17], [18]. Nonetheless, high levels of monitoring continued throughout most of this period, and the gorilla population continued to increase, reaching 380 individuals in 2003. Approximately 70% of that population is now habituated for ecotourism or research [18]. Nearly 20,000 tourists visited habituated groups in Rwanda in 2008, generating approximately $8 million in revenue for the park service and providing local employment [19]. Despite these benefits, habituation potentially increases the risk of disease transmission between humans and the gorillas [20], [21].

To protect the gorillas from poaching threats, the Virungas currently have more than 50 field staff per 100 km^2^, which consists of both national park and NGO staff, and is more than 20 times the global average [12]. The staff is primarily funded by revenue generated through ecotourism and NGOs, with the latter revenue being especially critical during times of military conflict. The staff patrols the entire park and confiscates more than 1500 snares per year. In addition, each habituated gorilla group is now continuously guarded by a separate team of field staff during daylight hours. To reduce the threat of disease transmission, tourists and researchers are required to stay at least seven meters away from the gorillas, but adherence to this rule can be difficult due to dense vegetation and the behavior of the gorillas. The veterinary program provides an additional line of protection against both threats by treating habituated gorillas for snare wounds, respiratory diseases, and other life threatening conditions.

Efforts to save the Virunga mountain gorillas represent an exceptional opportunity to compare two different conservation approaches in the same population at the same time. While the entire population has received conventional conservation measures such as ranger patrols and law enforcement, habituated gorillas have also received the more extreme approaches of continuous monitoring and *in situ* veterinary care. We use time-series analyses to compare the growth rates of the habituated versus unhabituated gorillas, as well as Leslie matrix calculations and individual-based models to provide more detailed results for the habituated groups. For example, we quantify how the growth rate of habituated groups has been influenced by poaching, respiratory disease, and veterinary interventions. In addition to examining those anthropogenic factors, we considered whether differences in the growth rates arose from socioecological influences such as feeding competition. If so, then we might expect lower reproductive success for females in larger groups and areas of lower food density [22]. We discuss the broader implications of our results for optimizing the conservation of other critically endangered species.

## Results

### Calculations of the actual growth rate

The habituated groups have grown from 30 gorillas in 1967 to 339 gorillas at the end of 2008 (diamonds in Figure 1a), which represents an average increase of 6.6% per year. However that increase partially reflects the habituation of 122 additional gorillas throughout the study period, as well as dispersal between the habituated and unhabituated groups (Text S1; Table S2). After adjusting for all exchanges between the habituated and unhabituated groups (Text S1, Section C), time-series analyses indicate an average growth rate of 4.1%±0.088% SD per year for habituated gorillas (See the Methods and Text S1, Section C for details about how we adjusted for exchanges between the habituated and unhabituated groups.) After adjusting for the exchanges, the time series analyses indicate that unhabituated gorillas had an average growth rate of −0.7%±0.059% (Figure 1b). Habituated gorillas had a higher growth rate than unhabituated gorillas in all five intervals between consecutive censuses (paired t-test: t = 4.1, df = 4, p = 0.015; Text S1, Section C).

**Figure 2:**
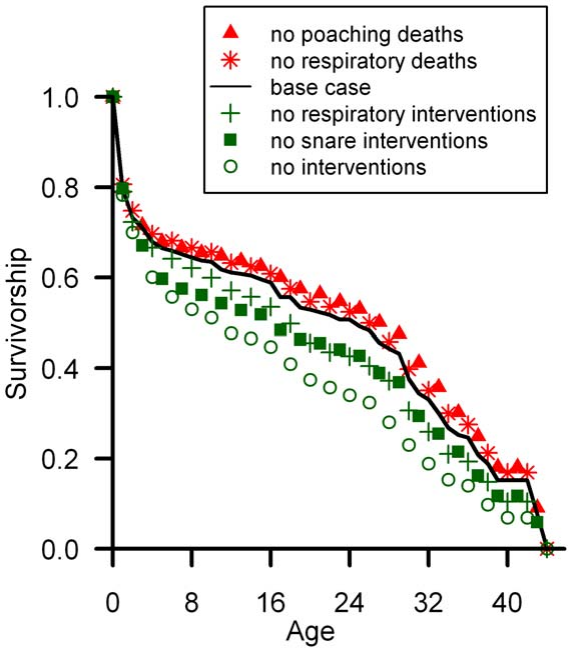
Survivorship curves used in Leslie matrix models for the growth rate of habituated groups. Listed from top to bottom, the cases show survivorship without poaching deaths (red triangles), without respiratory deaths (red asterisks), the base case (i.e., the complete dataset, black line), without veterinary interventions for respiratory disease (green plus marks), without interventions for snare wounds (green squares), and without any veterinary interventions (green circles). These values represent the modeling with 100% mortality in the absence of the veterinary interventions; assuming lower mortality would move the lines closer to the base case model.

A Leslie matrix model predicts a growth rate of 3.1% in the habituated groups (Text S1, Section B) based on an average rate of 0.255 births per adult-female year and age-specific survivorship values (Figure 2). Whereas the time-series analyses quantify how a population size has changed in the past, the Leslie matrix calculations predict what the growth rate would be if the population maintained the specified survivorship and birth rates for several generations [23]. The Leslie matrix calculations assume that exchanges between the habituated and unhabituated groups would become negligible in the long-term. We used the age-based Leslie matrix models to calculate the elasticity of the growth rate to survival and fertility as a function of age (Figure S6). Elasticity is defined as the percentage change in a model output (variable) relative to a percentage change in model parameter [24], [25]. Fertility accounts for only 5% of the overall elasticity in the growth rate, with another 42% coming from survival of immatures, and the remaining 53% from adult survival. Those results suggest that the population growth rate is more sensitive to proportional changes in survivorship than fertility.

Even if the habituated and unhabituated gorillas faced the same socioecological and anthropogenic influences, their fertility and mortality rates could differ due to demographic stochasticity. To estimate the potential magnitude of such demographic stochasticity in the habituated groups, we converted the Leslie matrix model into an individual-based dynamic model. We ran 1000 simulations of the habituated population, which showed a standard deviation of only ±0.4% among the 1000 growth rate predictions (with a mean growth rate of 3.9%). Even less demographic stochasticity would be expected for the unhabituated groups, because on average during the course of the study, their subset of the population had more gorillas (195±20 SE among the censuses) than the habituated groups (123±37). Thus demographic stochasticity could explain only a small portion of the difference in growth rates between the habituated versus unhabituated gorillas.

### Estimates of socioecological influences upon the growth rate

**Figure 3:**
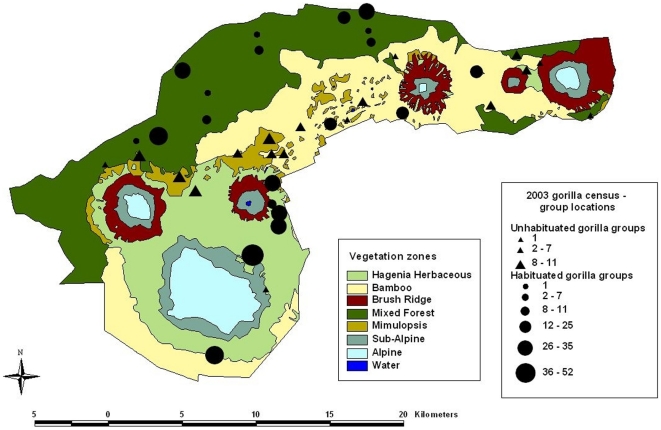
Distribution of the Virunga mountain gorilla groups in the 2003 census, and satellite mapping of vegetation zones throughout their habitat. The sizes of the circles and triangles indicate the total number of gorillas in each habituated and unhabituated group [18]. The dry weight biomass of foods consumed by gorillas was 74.3 g/m^2^ for hagenia and herbaceous zones, 4.2 g/m^2^ for bamboo, 15.4 g/m^2^ for brush ridge, 19.3 g/m^2^ for mixed forest, 18.8 g/m^2^ for mimulopsis, and 25.0 g/m^2^ for subalpine zones [35]. The alpine and water zones are not gorilla habitats.

The Virunga Massif is an afro-montane forest varying in altitude from 1500–4500 m, with several habitat types that differ in the biomass density of foods consumed by the gorillas (Figure 3). Using data from vegetation sampling, satellite imagery, and home range utilization, we calculated that the average food density varied from 4.2 to 66.3 g/m^2^ among habituated groups. The sizes of those groups have varied from 2–65 individuals. Despite the wide variations in those factors, we found no significant evidence that female reproductive success was limited by food availability or the assumed increased energetic demands of large group size (Table 1). Given that group sizes and food density for unhabituated groups fall within the ranges for habituated groups, those factors do not appear to account for differences in growth rates between the two populations.

**Table 1:** Generalized linear mixed models for the potential effects of group size and biomass density upon the age of first parturition (P_1_), offspring survival (I_SURV_), and interbirth intervals with offspring that survive to reach weaning age (IBI).

Dependent	Independent					standard		
variable	variable	N_DATA_	N_MOM_	N_GRP_	coefficient	error	t	P
P_1_	Group size	52	52	14	−0.004	0.016	−0.222	0.537
P_1_	Biomass	52	52	14	0.002	0.012	0.157	0.869
I_SURV_	Group size	276	110	19	−0.003	0.016	−0.204	0.838
I_SURV_	Biomass	276	110	19	−0.003	0.009	−0.321	0.748
IBI	Group size	133	69	18	−0.196	0.083	−2.373	0.059
IBI	Biomass	133	69	18	0.027	0.052	0.516	0.748

The identity of the group was included as a random effect in all analyses. The identity of the mother was included as a random effect in the analyses of I_SURV_ and IBI, but not P_1_ because that analysis involved only one data point per mother. N_GRP_, N_MOM_, and N_DATA_ are the number of groups, mothers, and total data points involved in each analysis. Although the relationship between group size and IBI is nearly significant, it is in the opposite direction of predictions for feeding competition. No significant results emerged from multivariate analyses, including when we added an interaction term (biomass/group size).

In addition to feeding competition, two other socioecological influences upon primates are predation and infanticide [22]. The Virunga mountain gorillas currently have no natural predators, and we estimated that infanticide has not been a major source of variability in their population growth rate (Text S1, Section E). Therefore, we conclude that socioecological factors have made little or no contribution to variability in the population growth rate, so differences between the habituated versus unhabituated gorillas were more likely to arise from human influences.

### Estimates of human impacts upon the growth rate

Throughout this study, 26 habituated gorillas have been killed by humans, representing 12% of all known mortality in this study. The time-series analyses indicate that if no gorillas had died from poaching, the growth rate in habituated groups would have been 4.6%±0.069% SD (Table 2). Three of the poaching deaths were due to gorillas getting caught in snares set for other animals, fifteen died as a result of shootings by militia groups, and eight were killed by villagers or poachers for various reasons including to capture gorillas for the pet trade, to stop crop raiding, or for bushmeat. Sixteen habituated gorillas died from respiratory disease during this study, but it is unknown whether those diseases were transmitted from humans, and the overall prevalence of human borne infections in gorillas was not quantified [26]–[28]. If no gorillas had died from respiratory disease, the growth rate of the habituated groups would have been 4.5%±0.072% (Table 2).

**Table 2:** Time series analyses and Leslie matrix models for the growth rate of habituated groups.

			Time	Leslie
			series	matrix
	N	Rate ± SE	analyses	models
Base case			4.1%	3.1%
Excluding deaths from:				
Poaching	26	0.0046±0.00023	4.6%	3.5%
Respiratory disease	16	0.0028±0.00021	4.5%	3.3%
Assuming death without veterinary interventions for:				
Snare wounds	42	0.0074±0.00034	3.4%	2.4% [2.8%]
Respiratory disease	42	0.0074±0.00022	3.4%	2.5% [2.8%]
Other	28	0.0050±0.00014	3.7%	2.8% [3.0%]
All	112	0.0198±0.00051	2.2%	1.4% [2.3%]

The base case used the actual data for survivorship and fertility throughout the study, without making any adjustments for any types of deaths or veterinary interventions. The additional cases excluded deaths from poaching or respiratory disease, and added deaths when gorillas received veterinary interventions. The rate per gorilla-year equals the number of those deaths or interventions (N), divided by the 5652 gorilla-years observed during this study. The standard errors (SE) for those rates are calculated among the calendar years observed. Numbers in brackets indicate Leslie matrix predictions of the growth rate if 50% of the gorillas treated would have died in the absence of veterinary treatment. See the Methods for estimates of other potential sources of uncertainty in the growth rates.

Forty two interventions were conducted to treat snare wounds on habituated gorillas. All but one of those individuals survived, but if treatment had not been available and they had died, the predicted growth rate for the habituated groups would have dropped from 4.1%±0.088% to 3.4%±0.066% (Table 2). Veterinarians monitored seventeen outbreaks of respiratory disease affecting more than 245 gorillas in the habituated groups (Table S3; Text S1, Section H). Forty-two gorillas were treated and 36 recovered (86%). If all 42 gorillas had died, the growth rate in habituated groups would have been 3.4%±0.068% SD.

**Figure 4:**
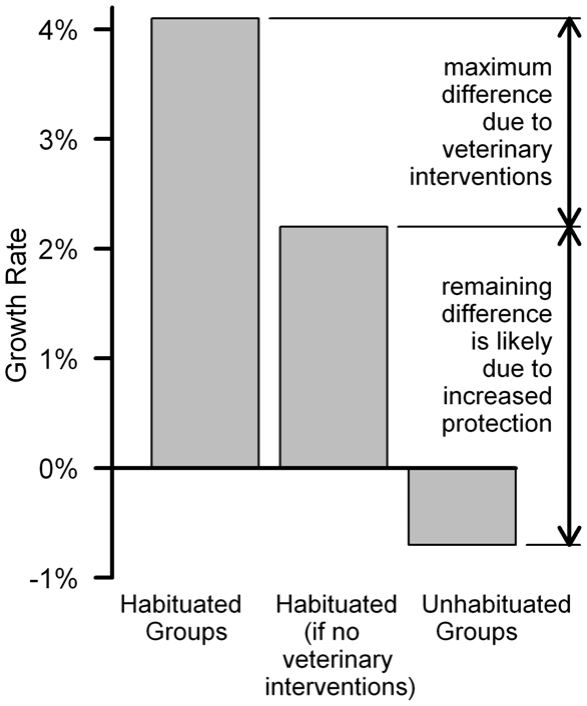
Time series analyses of growth rates under different management regimes. The first bar shows the growth rate for habituated groups, which received both continuous monitoring and veterinary treatment. The second bar estimates what the growth rate would have been for habituated groups, if they had been monitored continuously but did not receive veterinary care, and all afflicted gorillas had died instead. The difference between the first bar and second bar represents the maximum potential impact of veterinary interventions on the habituated gorillas (assumption of 100% mortality without interventions). The third bar shows growth rate for the unhabituated groups, which did not receive either continuous monitoring or veterinary treatment. The difference between the second bar and third bar shows what the impact of continuous monitoring could have been without veterinary interventions. Most of this difference was likely to arise from increased protection against poachers.

Additionally, the veterinarians have treated 28 habituated gorillas for other injuries and illnesses. If veterinary treatments had not been available, and all of the gorillas afflicted by snares, respiratory disease or other maladies had died instead, the time-series analyses indicate a growth rate of 2.2%±0.069% for the habituated groups (Table 2). That growth rate would have been higher if some gorillas could have survived without interventions (e.g., see Text S1, Section D, Figure S2, and the values in brackets in (Table 2), but treatment is typically withheld until conditions are considered life-threatening (Text S1, Section H). Thus, the time series analyses of habituated and unhabituated gorillas suggest that veterinary interventions could account for up to 40% of the difference between their growth rates (Figure 4).Given that demographic stochasticity and socioecological factors showed little or no influence on growth rates, we attribute the remaining difference (60% or more) between the habituated and unhabituated populations as resulting mainly from the increased protection provided by daily monitoring.

## Discussion

This study is one of the most comprehensive investigations of factors influencing the growth rate of an endangered primate, made possible through intensive long-term management, monitoring and research [17], [29], [30]. In contrast with the sharp declines of other great ape populations, the Virunga mountain gorillas have sustained a 1% growth rate over the past four decades (Figure 1), but habituated gorillas have been growing at a higher rate than unhabituated gorillas (4.1%±0.088% growth versus 0.7%±0.059% decline per year). Detection and veterinary treatment of illness/injury could account for up to 40% of the difference between the habituated versus unhabituated groups (Figure 4), so most of the remaining difference (60% or more) was likely to arise from increased protection against poachers. Therefore, daily monitoring of each habituated group contributed to most of the difference in growth rates, because it increased protection against poachers and it facilitated the veterinary program by spotting the ailments that have been treated.

Using data from long-term research, elasticity analyses suggest that conservation efforts should place more emphasis on improving survivorship than fertility, because fertility has less impact on population growth [31]–[34]. This conclusion is also supported by our results showing that conservation efforts to improve biomass density would have little or no impact upon female reproductive success (Table 1). The apparent lack of significant feeding competition over a wide range of habitats may suggest that all of the gorillas have relatively abundant food, and that the population is below their carrying capacity [35], [36]. However some herbivores may not show evidence of density dependence until their ecosystem is highly altered and damaged [37], [38], so careful monitoring of the gorilla habitat is warranted.

Concerns about the habituation of primates have increased in recent years because habituated animals can be more vulnerable to poachers and face greater risk of disease transmission [39], [40]. Habituation and research have provided a detailed understanding of the gorillas that has been instrumental for the successful development of the ecotourism program [41]. Given their precariously small population, the mountain gorillas remain vulnerable to epidemics and armed conflicts, both of which have decimated other gorilla populations [39], [42]. Habituated Virunga gorillas have occasionally been directly targeted and killed by humans, but the overall population was spared from far greater losses during the civil unrest because the gorillas are not traditionally eaten as bushmeat and because the economic value of ecotourism was recognized by the local communities and all parties involved in the political conflicts [17]. To reduce killing of gorillas, anti-poaching patrols should be improved, particularly in areas where the unhabituated groups range, and community-based programs should be expanded [4], [43]. To minimize the threat of human pathogens and reduce the need for veterinary interventions, rules for visiting the habituated gorillas should be strengthened [44] and some gorilla groups should remain unhabituated (Text S1, Section I). Extreme conservation and more conventional approaches are both essential for maximizing the long-term growth of the Virunga mountain gorillas.

This study suggests that ecosystem-based conservation strategies are necessary, but may not be enough to prevent some population declines, even when intensively applied in a relatively small area. Habituation and close monitoring of primates is typically done for research purposes and this study is consistent with other reports of their benefits for conservation [45], [46]. Disease management of endangered wildlife has often involved immunizations, rather than the cure of infected individuals as described in this study [47], [48]. Additional extreme conservation approaches include the creation of habitat corridors to link isolated populations [49], modifying existing habitat (e.g., bridges and tunnels around roadways) [50], removal of predators or exotic species [51], ecotourism [52], provisioning [53], translocations [54], and at the most extreme, captive breeding [55]. The results of this study argue for wider consideration of extreme conservation measures in addition to ecosystem based approaches, but the optimal approach for saving each species will depend upon its socioecology, its population size, and the specific threats to its survival.

Extreme conservation methods will generally be more practical to implement when the remaining populations are small, terrestrial, the animals have relatively small home ranges, and they are relatively easy to locate. Similar to ecosystem-based conventional approaches, *in situ* extreme measures may benefit not only the target species, but indirectly improve conditions for other species living in the same habitat and for people living in the surrounding communities. In contrast, however, extreme conservation is more likely to alter the natural behavior or life history of a species, potentially disrupting natural selection by helping less fit individuals to survive, and even leading to new threats such as human induced disease [47], [56]. Given these issues, it is necessary to evaluate whether it is more strategic to increase the intensity of conventional ecosystem-based approaches or develop extreme methods, further emphasizing the need to monitor the effectiveness of strategies applied [1], [2].

The implementation of most conservation programs has been limited by resources, and extreme measures can require more money and manpower than conventional approaches. The relative cost effectiveness of both approaches could influence the optimal distribution of conservation resources among and within species [57]. In a world where resources for conservation are finite, the channeling of resources toward one species is unavoidably done to the detriment of the conservation for other species. Until sufficient money is made available, conservationists will continue to be faced with the dilemma of devoting more resources to save a few species versus spreading resources too thinly to achieve success with any species [58]. Similarly, when focusing on a single population, spreading resources too thinly over a large area may reduce the likelihood of saving even a small area [59], [60]. Conservationists even face trade-offs about whether to divert limited resources away from direct conservation activities in order to perform rigorous cost benefit analyses of their effectiveness [1]. The call for more resources is justifiably common [3], [61], but if conventional ecosystem-based measures cannot succeed alone, then the need for additional resources could be far greater than typically anticipated. The extraordinary efforts needed to save the mountain gorillas may imply sobering prospects for some endangered species, but our results argue for the continued development of creative, cost-effective, and efficient approaches to conservation.

## Methods

### Ethics Statement

This research involved non-invasive work with wild non-human primates. All work was done in accordance with guidelines of the national authorities where the work occurred.

### Study population

The size of the overall population (and the unhabituated gorillas) was measured during six censuses of the entire region from 1971–2003 (Figure 1a and Figure S3) [18]. Additional demographic data for births, deaths, and dispersal patterns of 668 gorillas are reported from 20 social units (groups and solitary males) that have been habituated by the Dian Fossey Gorilla Fund International Karisoke Research Center^g^ since 1967, along with 26 social units that have been habituated for tourism and monitored through the Ranger Based Monitoring programs of the three national park services (Text S1, Section A; Table S1). Gorillas are naturally afraid of humans and ‘habituated’ means that through repeated, neutral contact with humans, they exhibit normal behavior when people are in close proximity.

### Overview of growth rate calculations

We examined the population growth rates from three perspectives: time series analyses (Text S1, Section C), Leslie matrix calculations (Text S1, Section B), and a dynamic individual based model (Text S1, Section D). The time series analyses provided the main basis for comparing the growth rates of habituated versus unhabituated gorillas, because it did not require data for vital rates (mortality and fertility) which were not available for unhabituated gorillas. We used Leslie matrix calculations to provide a longer-term perspective on the growth rate of habituated gorillas, and for elasticity analyses, and for future comparisons with other studies. We used the individual based dynamic model to evaluate demographic stochasticity and temporal variability in the age/class structure of habituated gorillas (Figure S3).

### Leslie matrix calculations of the growth rate

We used Leslie matrix models to predict what the growth rate of the habituated groups would be if they maintained the specified mortality and fertility rates for several generations and equilibrated into a stable age distribution (Text S1, Section B). Mountain gorillas are not seasonal breeders so we used birth flow calculations, as in pages 23–25 of [23]. Each year of age was a separate stage in the models. Mortality probabilities (Q) for each age (x) were calculated as the number of deaths divided by the number of gorillas that reached that age (gorilla-years started). The survival probability (P_x_) equaled 1 – Q_x_. Survivorship (L) to reach age x was calculated as the product of P_x_ from all preceding ages (Figure 2 and Figure S1).

To simulate what the growth rate would be without deaths from poaching or respiratory disease, we removed those reported deaths from the survivorship data. Data was censored at the age when the individual was last observed. To simulate what the growth rate would be without veterinary interventions, we added a death at each age when each of those interventions occurred. For example, if a female received veterinary treatment at age 20, but survived until age 30, we added another death to the life table at age 20, while retaining the subsequent data for the female. Results were similar when we removed all subsequent data for individuals after their treatments. For sensitivity studies of the impact of veterinary interventions, we adjusted the mortality probability for the ages when those events occurred, with the assumption of 25, 50, 75, and 100% mortality in the absence of veterinary care (Text S1, Section D; Figure S2).

### Time series analyses of the growth rate

We used time-series calculations to quantify what the growth rate has actually been for both habituated and unhabituated groups, during each decade and throughout the entire study (Text S1, Section C). The growth rate was determined by starting with an initial number of gorillas and using Equation 1 to calculate the number of gorillas in each subsequent month:



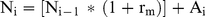
(1)In that equation, N_i_ represents the number of gorillas in month “i”, N_i-1_ is the number of gorillas in the previous month, r_m_ is the monthly growth rate. The adjustment factor “A_i_” equaled the number of gorillas that joined the specified groups during each month (e.g. through immigration or additional habituation), minus the number of gorillas that left those groups (e.g. through emigration). The time series analyses include both males and females, but the values for A_i_ do not account for the age or sex in which each adjustment occurred (Table S2). We used iterative calculations with the bisection method to find the value of r_m_ that enabled us to match the observed size of the habituated groups at the end of the study period [62]. The monthly growth rate was converted into an annual growth rate (r_a_) using Equation 2, to account for monthly compounding.



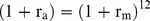
(2)To simulate what the growth rate would have been without deaths from poaching or respiratory disease, we subtracted the count of those deaths from the value of A_i_ in the year/month when they occurred. For example, if a poaching death occurred in December of 1974, we reduced the value of A_i_ by one gorilla for that year/month. To simulate what the growth rate would have been without veterinary interventions, we added the number of those interventions to the value of A_i_ in the month when they occurred.

The overall population growth rate for unhabituated gorillas had an estimated standard deviation of ±0.12% due to uncertainty in their census counts (Figure S7), and a standard deviation of ±0.059% due to uncertainty in the fate of gorillas that disappeared from habituated groups^c^. The convergence tolerance of the bisection method caused less than 0.0005% uncertainty in the estimated growth rates of habituated and unhabituated gorillas^c^. The number of habituated gorillas was known exactly due to direct daily observations, so their growth rates had essentially no uncertainty from census counts. In Table 2, the “base case” population growth rate for habituated gorillas had an estimated standard deviation of ±0.088% due to uncertainty in the fate of gorillas that disappeared from their groups. Unless otherwise stated, the main text shows standard deviations that are based on uncertainty in the fate of gorillas that disappeared from habituated groups, but those results should be considered a lower limit for the overall uncertainty in the growth rates from time series analyses (Text S1, Sections C, D, J).

### Individual-based dynamic model of demographic stochasticity

To estimate the potential magnitude of demographic stochasticity in the habituated groups, we converted the Leslie matrix model into an individual-based dynamic model, as in Section 15.1.2 of [23]. The initial group compositions (and subsequent habituation and dispersal) were fixed to match the age/sex classifications of the actual gorillas entering and leaving the habituated groups in each year. The model tracked each individual through the years of the study, using a random number generator for values between 0–1. For example, if a gorilla had survival probability (P_x_) of 0.95 in a particular year of the simulation, then it survived until the next year unless the randomly generated number was greater than 0.95. The model generated a new random number each time it evaluated whether a gorilla would give birth or die in each year. The model included separate mortality data for adult males (to simulate the entire age/sex structure), but we assumed that males had no influence upon the birth rate. We used Equations 1 & 2 to convert the final number of gorillas into an annual growth rate.

### Female reproductive success versus group size and biomass density

We used published data from vegetation sampling for the average biomass density of the home ranges of the research groups studied prior to 1993 [35], [63], [64]. We used three sets of data to determine the average biomass density for the home ranges of most groups monitored in the 1990s and 2000s (Text S1, Section E). First, we used previously reported data from vegetation sampling for the average biomass density of six vegetation zones (see the Legend in Figure 3). Second, we used data from satellite imagery to determine the distribution of those six vegetation zones throughout the Virungas (Figure 3). Third, we estimated the home range of each group using GPS points of their daily locations throughout 2004. The average biomass density of a group equaled the proportion of time that the group spent in each vegetation zone, multiplied by the biomass density for each respective zone. Detailed ranging data was not available for four groups so we assumed that their biomass density was similar to other groups whose home range had a similar location.

Analyses for the age of first parturition were limited to data points in which the age of the mother and her first offspring were both in known to within 15 days (Figure S4). Similarly, the analyses of interbirth intervals (IBI) were limited to data points in which the beginning and end of the interval was known to within 15 days. The dependent variable for offspring survival equaled “1” when an offspring survived to reach age three, and “0” when it did not. Analyses of offspring survival were limited to data before 2006, because the study ended before we could fully evaluate the survival of subsequent offspring. To focus on the potential effects of feeding competition, the analyses of offspring survival also excluded infants that were killed by poaching or infanticide (Figure S5). The analyses of IBI and offspring survival do not include primiparous mothers, who have shown lower reproductive success than multiparous females [65].

Analyses of offspring survival were done using generalized linear mixed models (GLMM) with a binomial error distribution, by specifying that “family = binomial” in the “lmer” function of the “lme4” package developed for R (Version 2.7.0, R Development Core Team 2008, http://www.R-project.org). Analyses of IBI and the age of first parturition were also done using GLMM, but with a Gaussian error distribution because the response variable was continuous rather than dichotomous. The lmer function does not report p-values for analyses with a Gaussian error distribution, so we estimated those p-values using a bootstrap procedure with 10,000 iterations.

## Supporting Information

Figure S1 **Survivorship curves for male (triangles) and female (circles) mountain gorillas, depending upon whether unexplained disappearances were due to dispersal (filled symbols with lines) or deaths (open symbols without lines).**
(TIF)

Figure S2 **Predicted growth rates for all habituated groups if gorillas had died instead of receiving veterinary care for snares (triangles), respiratory diseases (circles), both (plus-marks), “other” (squares, see** **Methods** **for which interventions are included in this category), or all three categories of interventions (x-marks).** The x-axis represents the assumed probability that a gorilla would have survived to complete the year of age in which it received such veterinary care, if the care had not been provided.
(TIF)

Figure S3 **Proportion of immature gorillas (black circles), adult females (red triangles), and adult males (blue squares) in the habituated groups.** Solid lines are the average values from 1000 simulations with the individual-based dynamic model. The dashed lines represent the stable age structure that would arise if survivorship and fertility remained fixed for several generations without exchanges between the habituated versus unhabituated groups.
(TIF)

Figure S4 **Quantile plots for the age of first parturition (circles), interbirth intervals when an offspring survives to reach age three (asterisks), and interbirth intervals when the offspring dies (triangles).** Sample sizes are 52, 133, and 73 respectively. Smoothed curves are from regressions of logit(quantile) versus ln(time).
(TIF)

Figure S5 **Survivorship curves for all 460 infants born during this study (black), and excluding six infanticide deaths during group disintegrations after the dominant silverback died (red), and excluding 31 deaths from poaching or known/suspected cases of infanticide (blue).**
(TIF)

Figure S6 **Elasticity of the growth rate to female fertility (triangles) and survival (circles) as a function of age.** In the main text, the elasticity for fertility equals the sum of the values at each age shown here. The elasticity for immature survival equals the sum of the values at each age from 0–7, and the elasticity for adult females equals the sum of the values from ages eight upward.
(TIF)

Figure S7 **Negative binomial distributions for the probability that a specified number of gorillas were missed in the 1972 census (triangles) and the 2003 census (diamonds).** For example, the negative binomial distribution function showed a 3% probability that exactly seven unhabituated gorillas were missed in the 1972 census.
(TIF)

Table S1 **Summary of the social units (groups and solitary males) that have been habituated for research (1a) and tourism (1b) in each country (Rw = Rwanda, DRC =  the Democratic Republic of Congo).** Some groups have ranged outside the country where they are listed. For example, Beetsme's group has ranged in both Rwanda and the DRC, and the Nyakagezi group has ranged in the DRC, Rwanda, and Uganda. First, last, and total years of observation for each group, as well as the proportion of months that the group was multimale (versus one-male). Number of gorilla-years and adult-female years observed, and total number of gorillas (average, minimum, and maximum) per group. Number of total births, and deaths, and unexplained disappearances (unex).
(DOC)

Table S2 **Summary of changes in the number of gorillas in habituated groups.** The immigrations and emigrations show only exchanges between the habituated and unhabituated groups, not among the habituated groups. The total number of changes (702) exceeds the total number of gorillas in the database (668) because some individuals have moved between the habituated and unhabituated groups more than once.
(DOC)

Table S3 **Respiratory outbreaks monitored by the Mountain Gorilla Veterinary Program (MGVP) between 1986 and 2008.**
(DOC)

Text S1 **Supporting Information.**
(DOC)
