# Supplementary figures and images for: Extreme Conservation Leads to Recovery of the Virunga Mountain Gorillas
Source: PLoS One. 2011 Jun 8;6(6):e19788. doi: 10.1371/journal.pone.0019788 (PMC3110611; doi:10.1371/journal.pone.0019788)

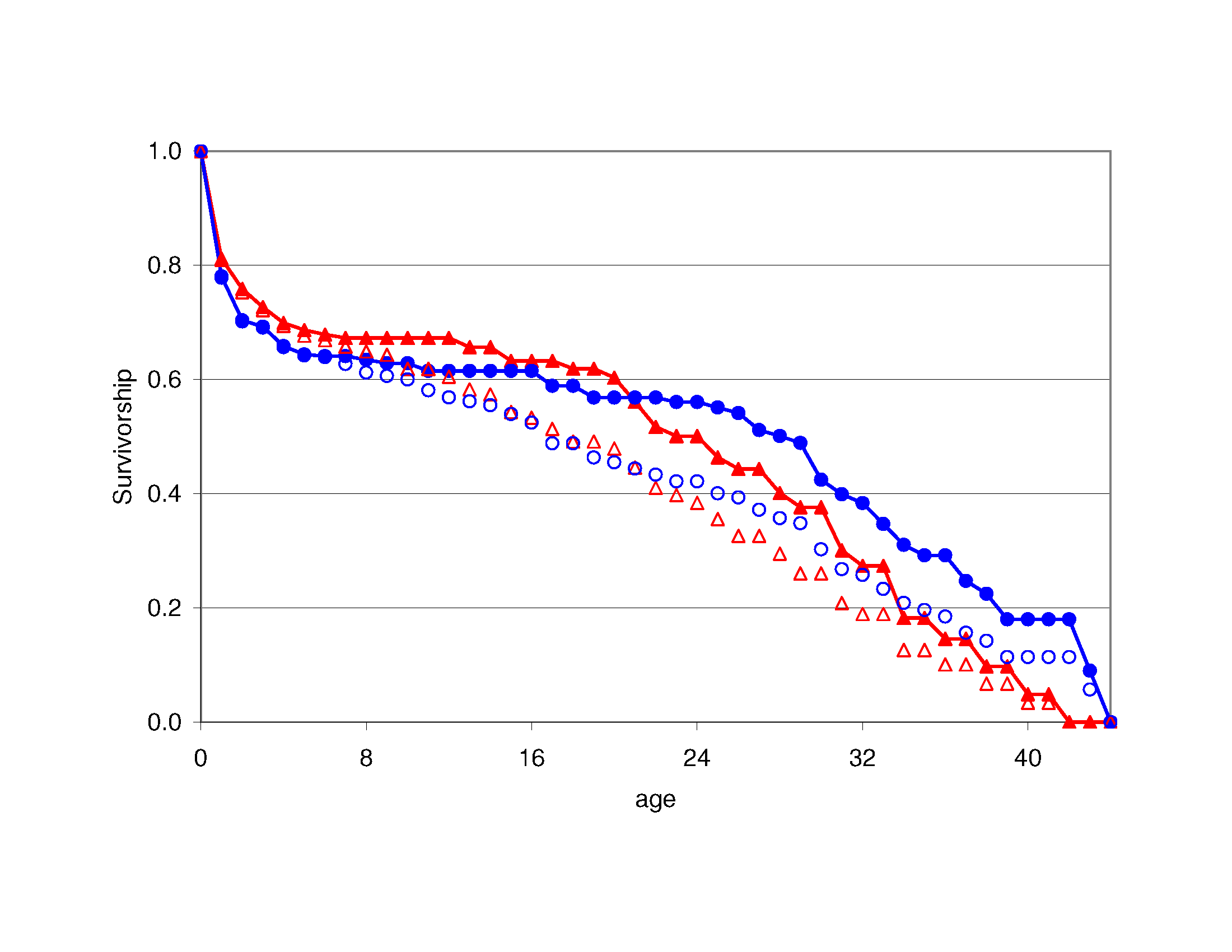

Supplement: Figure S1 — Survivorship curves for male (triangles) and female (circles) mountain gorillas, depending upon whether unexplained disappearances were due to dispersal (filled symbols with lines) or deaths (open symbols without lines). (TIF) [file pone.0019788.s001.tif]

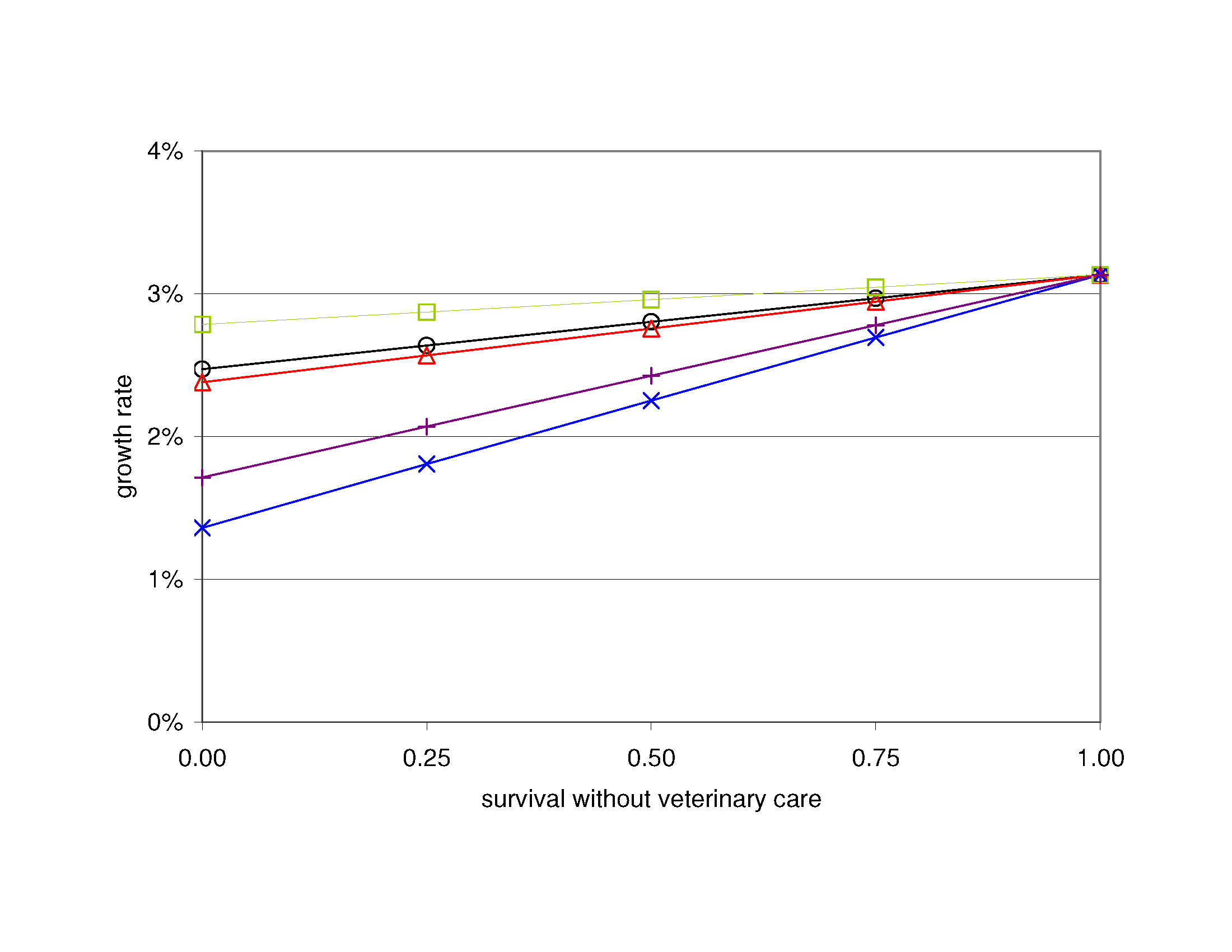

Supplement: Figure S2 — Predicted growth rates for all habituated groups if gorillas had died instead of receiving veterinary care for snares (triangles), respiratory diseases (circles), both (plus-marks), “other” (squares, see Methods for which interventions are included in this category), or all three categories of interventions (x-marks). The x-axis represents the assumed probability that a gorilla would have survived to complete the year of age in which it received such veterinary care, if the care had not been provided. (TIF) [file pone.0019788.s002.tif]

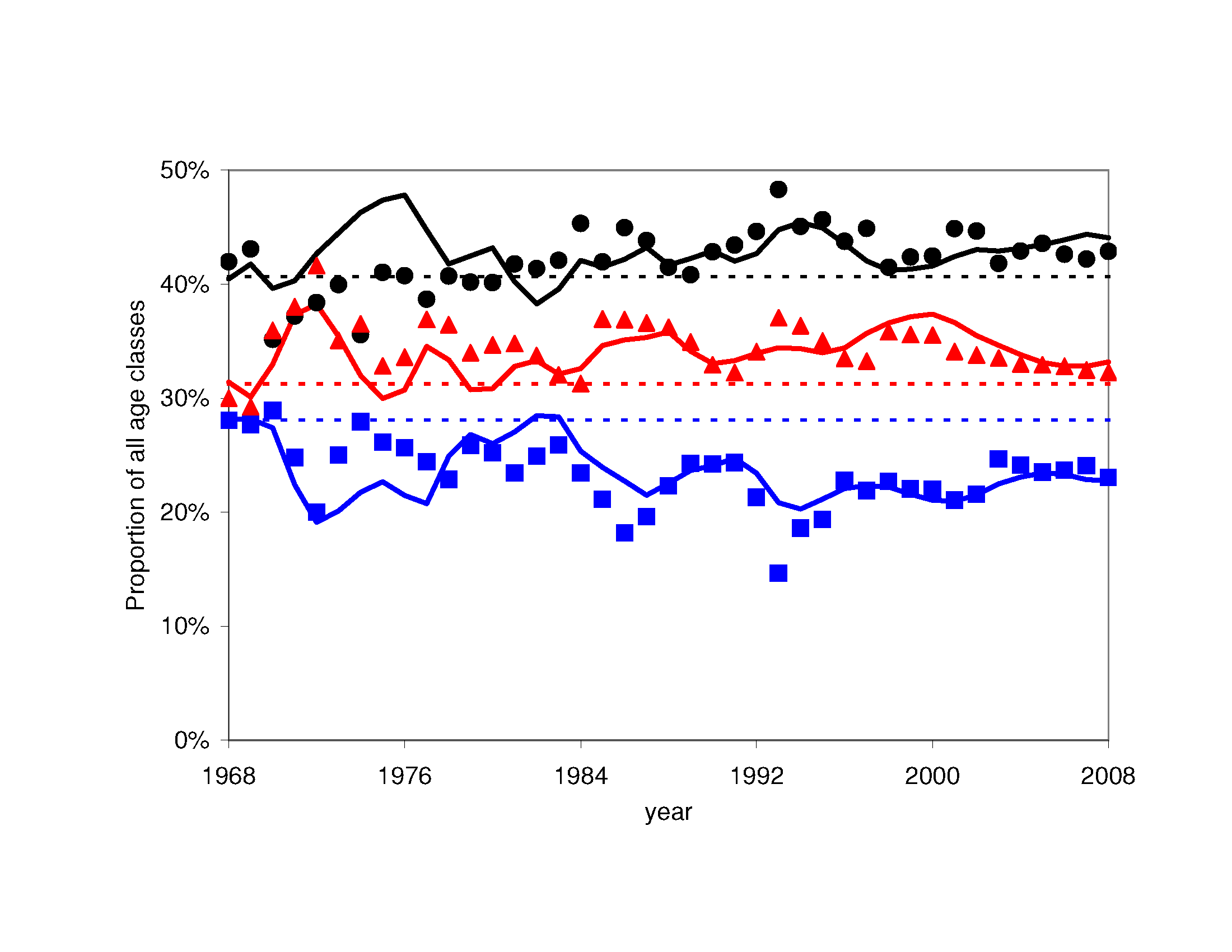

Supplement: Figure S3 — Proportion of immature gorillas (black circles), adult females (red triangles), and adult males (blue squares) in the habituated groups. Solid lines are the average values from 1000 simulations with the individual-based dynamic model. The dashed lines represent the stable age structure that would arise if survivorship and fertility remained fixed for several generations without exchanges between the habituated versus unhabituated groups. (TIF) [file pone.0019788.s003.tif]

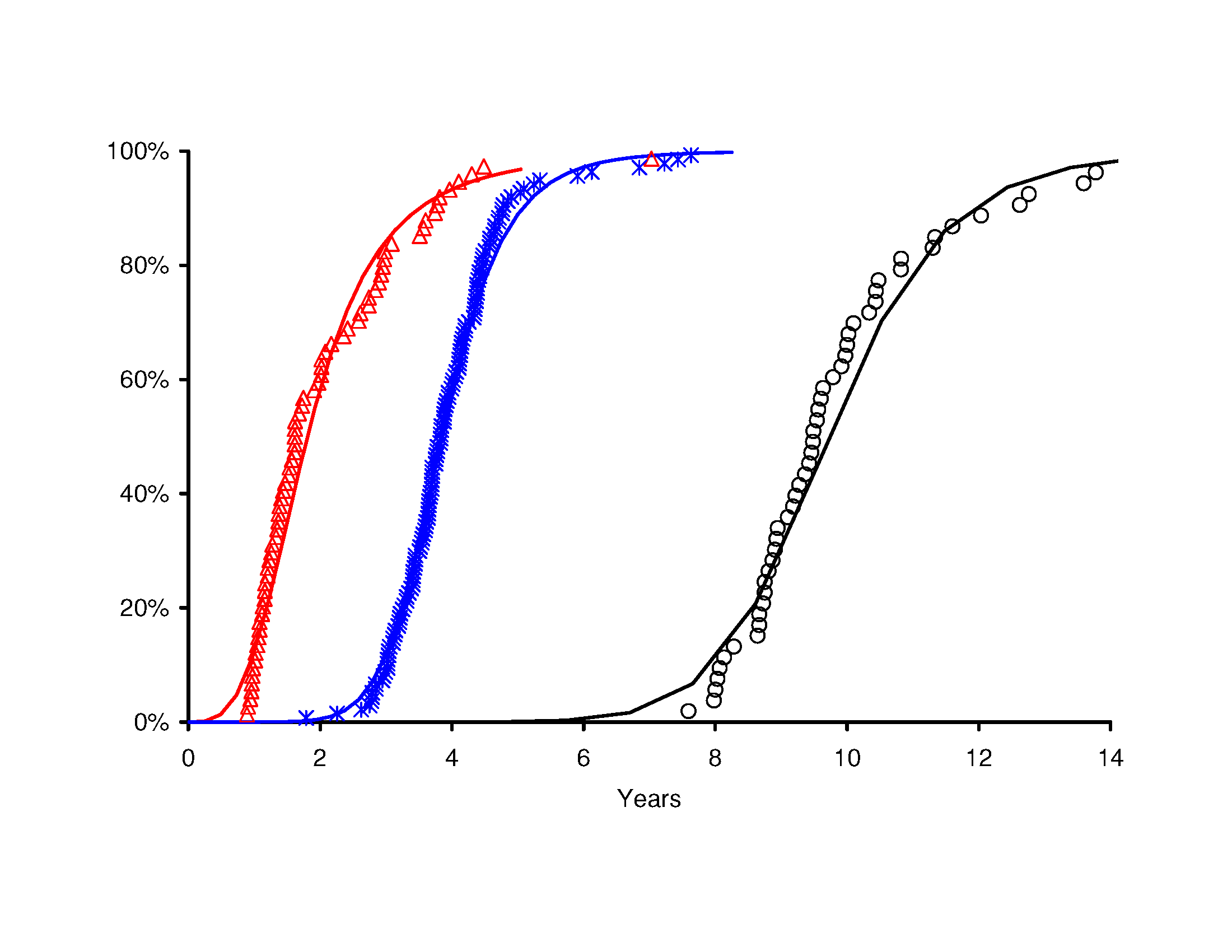

Supplement: Figure S4 — Quantile plots for the age of first parturition (circles), interbirth intervals when an offspring survives to reach age three (asterisks), and interbirth intervals when the offspring dies (triangles). Sample sizes are 52, 133, and 73 respectively. Smoothed curves are from regressions of logit(quantile) versus ln(time). (TIF) [file pone.0019788.s004.tif]

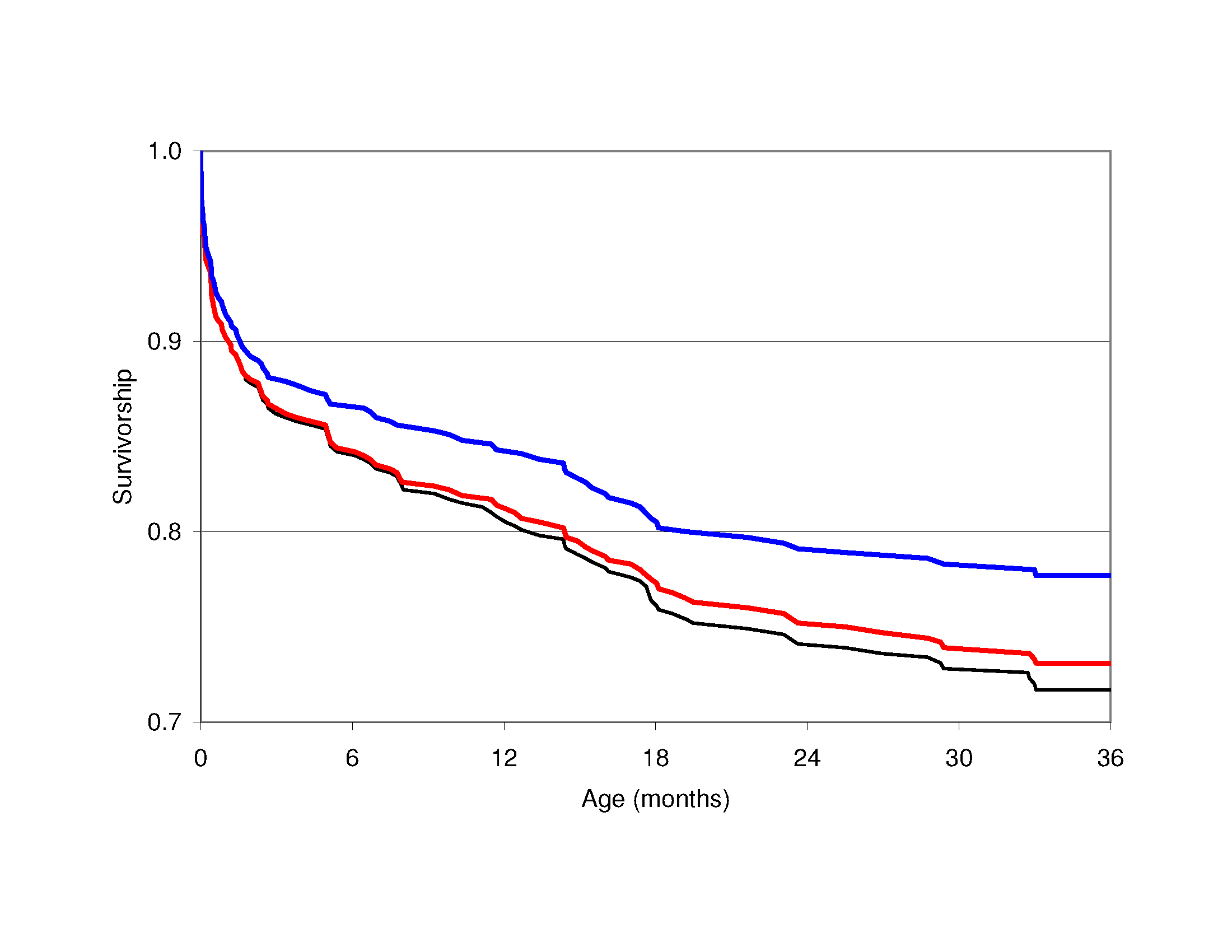

Supplement: Figure S5 — Survivorship curves for all 460 infants born during this study (black), and excluding six infanticide deaths during group disintegrations after the dominant silverback died (red), and excluding 31 deaths from poaching or known/suspected cases of infanticide (blue). (TIF) [file pone.0019788.s005.tif]

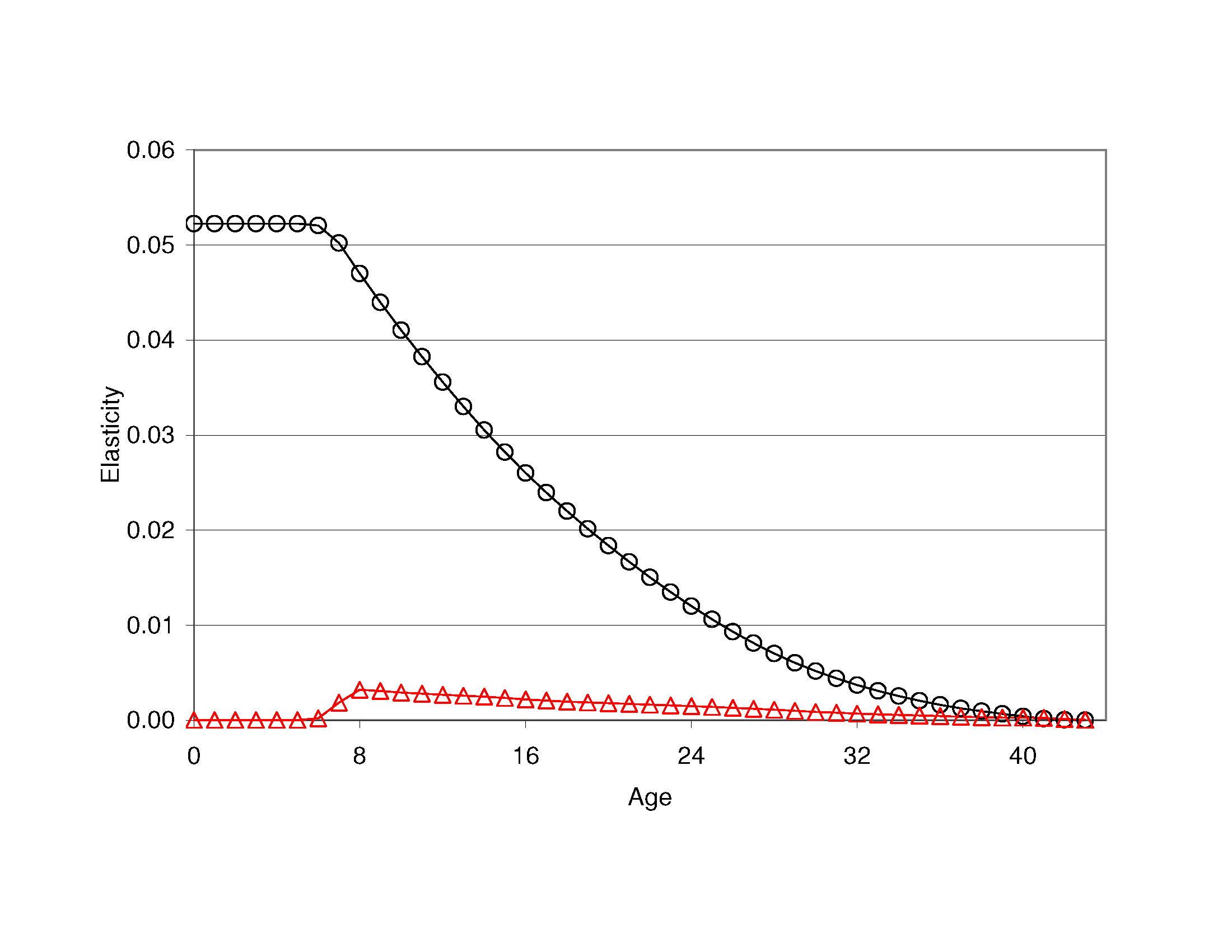

Supplement: Figure S6 — Elasticity of the growth rate to female fertility (triangles) and survival (circles) as a function of age. In the main text, the elasticity for fertility equals the sum of the values at each age shown here. The elasticity for immature survival equals the sum of the values at each age from 0–7, and the elasticity for adult females equals the sum of the values from ages eight upward. (TIF) [file pone.0019788.s006.tif]
