# Supplementary material for: Extreme Conservation Leads to Recovery of the Virunga Mountain Gorillas
Source: PLoS One. 2011 Jun 8;6(6):e19788. doi: 10.1371/journal.pone.0019788 (PMC3110611; doi:10.1371/journal.pone.0019788)
Supplement: Table S1 — Summary of the social units (groups and solitary males) that have been habituated for research (1a) and tourism (1b) in each country (Rw = Rwanda, DRC = the Democratic Republic of Congo). Some groups have ranged outside the country where they are listed. For example, Beetsme's group has ranged in both Rwanda and the DRC, and the Nyakagezi group has ranged in the DRC, Rwanda, and Uganda. First, last, and total years of observation for each group, as well as the proportion of months that the group was multimale (versus one-male). Number of gorilla-years and adult-female years observed, and total number of gorillas (average, minimum, and maximum) per group. Number of total births, and deaths, and unexplained disappearances (unex). (DOC) [file pone.0019788.s008.doc]

**Table S1.** Summary of the social units (groups and solitary males) that have been habituated for research (1a) and tourism (1b) in each country (Rw=Rwanda, DRC= the Democratic Republic of Congo). Some groups have ranged outside the country where they are listed. For example, Beetsme's group has ranged in both Rwanda and the DRC, and the Nyakagezi group has ranged in the DRC, Rwanda, and Uganda. First, last, and total years of observation for each group, as well as the proportion of months that the group was multimale (versus one-male). Number of gorilla-years and adult-female years observed, and total number of gorillas (average, minimum, and maximum) per group. Number of total births, and deaths, and unexplained disappearances (unex).

Table 1a. Social units habituated for research.

|  |  |  |  |  |  |  | adult |  |  |  | biomass |  |  |  |
| --- | --- | --- | --- | --- | --- | --- | --- | --- | --- | --- | --- | --- | --- | --- |
|  |  |  |  | group- | %multi | gorilla- | female | number of gorillas | | | density |  |  |  |
| Group | country | year1 | year2 | years | male | years | years | Avg | Min | Max | g/m2 | births | deaths | unex |
| Amok | Rw | 1969 | 1971 | 2.3 | 0% | 2.4 | 0.2 | 1.1 | 1 | 2 | ----- | 0 | 0 | 0 |
| Beetsme | Rw | 1985 | 2009 | 23.5 | 93% | 454.1 | 143.2 | 19.3 | 8 | 27 | 66.3 | 33 | 13 | 1 |
| Bwenge | Rw | 2006 | 2009 | 2.1 | 0% | 17.6 | 10.9 | 8.4 | 2 | 12 | 62.3 | 7 | 4 | 0 |
| Group 4 | Rw | 1967 | 1979 | 11.3 | 21% | 124.8 | 45.8 | 11.0 | 3 | 14 | 65.5 | 12 | 13 | 0 |
| Group 5 | Rw | 1967 | 1993 | 25.7 | 83% | 450.5 | 168.8 | 17.6 | 10 | 38 | 62.4 | 44 | 21 | 0 |
| Group 8 | Rw | 1967 | 1974 | 6.7 | 60% | 28.0 | 4.6 | 4.2 | 2 | 6 | 62.4 | 1 | 3 | 0 |
| Gwiza | Rw | 2004 | 2009 | 4.8 | 0% | 5.1 | 0.3 | 1.1 | 1 | 3 | ----- | 0 | 0 | 0 |
| Inshuti | Rw | 2004 | 2009 | 5.1 | 0% | 8.4 | 3.2 | 1.7 | 1 | 4 | ----- | 1 | 0 | 0 |
| Isabukuru | Rw | 2007 | 2009 | 1.6 | 0% | 9.8 | 4.8 | 6.2 | 4 | 7 | 62.3 | 2 | 1 | 0 |
| Kuryama | Rw | 2007 | 2009 | 1.5 | 100% | 18.8 | 7.3 | 12.6 | 9 | 14 | 62.3 | 3 | 0 | 0 |
| Nunkie | Rw | 1972 | 1985 | 12.6 | 0% | 119.5 | 53.6 | 9.3 | 1 | 18 | 66.1 | 15 | 7 | 1 |
| Pablo | Rw | 1993 | 2009 | 15.7 | 100% | 661.8 | 243.1 | 42.2 | 19 | 64 | 54.0 | 67 | 31 | 2 |
| PbSubgrp | Rw | 2007 | 2007 | 0.1 | 100% | 1.2 | 0.3 | 14.0 | 14 | 14 | ----- | 0 | 0 | 0 |
| Peanut | Rw | 1974 | 1992 | 17.3 | 55% | 88.5 | 0.0 | 4.8 | 1 | 7 | 66.3 | 0 | 1 | 0 |
| Samson | Rw | 1971 | 1976 | 4.6 | 0% | 7.3 | 2.8 | 1.6 | 1 | 2 | ----- | 1 | 1 | 0 |
| Shinda | Rw | 1993 | 2009 | 15.7 | 70% | 348.9 | 103.9 | 22.3 | 16 | 29 | 62.1 | 26 | 12 | 0 |
| Tiger | Rw | 1981 | 1987 | 6.1 | 0% | 9.3 | 2.1 | 1.5 | 1 | 3 | 62.1 | 1 | 2 | 0 |
| Turatsinze | Rw | 2006 | 2009 | 2.2 | 0% | 2.4 | 0.1 | 1.1 | 1 | 4 | 62.1 | 0 | 0 | 0 |
| Umurava | Rw | 2006 | 2006 | 0.1 | 0% | 0.4 | 0.0 | 5.0 | 5 | 5 | ----- | 0 | 0 | 0 |
| Umushikirano | Rw | 2006 | 2009 | 2.3 | 0% | 2.8 | 0.5 | 1.2 | 1 | 3 | ----- | 0 | 1 | 0 |

***Table S1b****. Social units habituated for tourism.*

|  |  |  |  |  |  |  | adult |  |  |  | biomass |  |  |  |
| --- | --- | --- | --- | --- | --- | --- | --- | --- | --- | --- | --- | --- | --- | --- |
|  |  |  |  | group- | %multi | gorilla- | female | number of gorillas | | | density |  |  |  |
| Group | country | year1 | year2 | years | male | years | years | Avg | Min | Max | g/m2 | births | deaths | unex |
| Amahoro | Rw | 1996 | 2009 | 12.8 | 62% | 200.5 | 75.4 | 15.6 | 12 | 19 | 50.5 | 14 | 6 | 2 |
| Buhanga | DRC | 1998 | 2009 | 10.9 | 0% | 11.2 | 0.3 | 1.0 | 1 | 2 | ----- | 0 | 1 | 1 |
| Bukima | DRC | 2007 | 2008 | 1.2 | 0% | 1.2 | 0.0 | 1.0 | 1 | 1 | ----- | 0 | 0 | 0 |
| Group11 | Rw | 1979 | 1993 | 13.6 | 53% | 138.0 | 33.0 | 10.2 | 6 | 15 | 19.5 | 11 | 19 | 12 |
| Group13 | Rw | 1979 | 2009 | 27.9 | 10% | 291.2 | 124.3 | 10.2 | 3 | 26 | 4.2 | 32 | 19 | 3 |
| Group9 | Rw | 1980 | 1992 | 12.4 | 0% | 102.1 | 36.2 | 8.2 | 6 | 11 | ----- | 6 | 12 | 6 |
| Hirwa | Rw | 2006 | 2009 | 2.6 | 0% | 26.6 | 12.9 | 10.3 | 8 | 12 | 4.2 | 4 | 0 | 0 |
| Humba | DRC | 1998 | 2009 | 10.5 | 37% | 106.3 | 36.2 | 10.1 | 9 | 12 | 11.9 | 6 | 3 | 0 |
| Kabirizi | DRC | 1997 | 2009 | 11.3 | 7% | 309.2 | 118.0 | 27.5 | 8 | 38 | 11.3 | 35 | 11 | 4 |
| Karateka | DRC | 1998 | 2009 | 10.9 | 0% | 10.9 | 0.0 | 1.0 | 1 | 1 | ----- | 0 | 0 | 0 |
| Karema | DRC | 2002 | 2007 | 4.6 | 0% | 4.6 | 0.0 | 1.0 | 1 | 1 | ----- | 0 | 1 | 0 |
| Kwitonda | Rw | 1998 | 2009 | 10.9 | 18% | 149.8 | 47.1 | 13.7 | 8 | 18 | 4.2 | 16 | 5 | 0 |
| Lulengo | DRC | 1998 | 2009 | 10.9 | 68% | 46.1 | 7.4 | 4.2 | 1 | 11 | 17.3 | 2 | 5 | 3 |
| Mapuwa | DRC | 1995 | 2009 | 13.3 | 6% | 102.7 | 51.6 | 7.7 | 1 | 15 | 13.3 | 11 | 4 | 1 |
| Mareru | DRC | 2006 | 2007 | 0.9 | 0% | 0.9 | 0.0 | 1.0 | 1 | 1 | ----- | 0 | 0 | 0 |
| Muguri | Rw | 1999 | 2009 | 9.3 | 0% | 9.3 | 0.0 | 1.0 | 1 | 1 | ----- | 0 | 0 | 0 |
| Munyaga | DRC | 1998 | 2009 | 10.9 | 47% | 72.8 | 9.4 | 6.7 | 4 | 12 | 11.8 | 3 | 3 | 0 |
| Mvuyekure | DRC | 2005 | 2008 | 2.6 | 0% | 2.6 | 0.0 | 1.0 | 1 | 1 | ----- | 0 | 0 | 0 |
| Nyakagezi | Rw | 1998 | 2009 | 11.0 | 100% | 97.6 | 23.8 | 8.9 | 7 | 11 | 4.2 | 5 | 3 | 2 |
| Nyakarima | Rw | 2003 | 2009 | 6.0 | 0% | 6.7 | 0.6 | 1.1 | 1 | 2 | ----- | 0 | 1 | 1 |
| PiliPili | DRC | 2002 | 2008 | 6.3 | 0% | 7.8 | 1.5 | 1.2 | 1 | 4 | 17.3 | 0 | 0 | 0 |
| Rugendo | DRC | 1997 | 2009 | 11.3 | 44% | 105.7 | 43.2 | 9.4 | 5 | 18 | 15.1 | 7 | 11 | 1 |
| Ruzirabwoba | DRC | 1995 | 2009 | 13.3 | 0% | 13.3 | 0.0 | 1.0 | 1 | 1 | ----- | 0 | 0 | 0 |
| Sabyinyo | Rw | 1989 | 2009 | 19.7 | 78% | 184.8 | 64.3 | 9.4 | 6 | 12 | 4.2 | 13 | 7 | 3 |
| Susa | Rw | 1978 | 2009 | 30.4 | 95% | 843.8 | 287.9 | 27.7 | 4 | 41 | 48.2 | 75 | 31 | 8 |
| Umubano | Rw | 2002 | 2009 | 6.7 | 0% | 47.5 | 17.8 | 7.1 | 4 | 9 | 60.3 | 5 | 1 | 0 |
