# Supplementary material for: Extreme Conservation Leads to Recovery of the Virunga Mountain Gorillas
Source: PLoS One. 2011 Jun 8;6(6):e19788. doi: 10.1371/journal.pone.0019788 (PMC3110611; doi:10.1371/journal.pone.0019788)
Supplement: Table S2 — Summary of changes in the number of gorillas in habituated groups. The immigrations and emigrations show only exchanges between the habituated and unhabituated groups, not among the habituated groups. The total number of changes (702) exceeds the total number of gorillas in the database (668) because some individuals have moved between the habituated and unhabituated groups more than once. (DOC) [file pone.0019788.s009.doc]

**Table S2**. Summary of changes in the number of gorillas in habituated groups. The immigrations and emigrations show only exchanges between the habituated and unhabituated groups, not among the habituated groups. The total number of changes (702) exceeds the total number of gorillas in the database (668) because some individuals have moved between the habituated and unhabituated groups more than once.

|  | In | Out |  |
| --- | --- | --- | --- |
| Initial number of gorillas | 30 | 339 | Final number of gorillas |
| Births | 460 | 211 | Deaths |
| Additional habituation | 122 | 4 | End of group monitoring |
| Immigration | 90 | 97 | Emigration |
|  |  | 51 | Unexplained disappearances |
| Total | 702 | 702 | Total |
