# Supplementary material for: Extreme Conservation Leads to Recovery of the Virunga Mountain Gorillas
Source: PLoS One. 2011 Jun 8;6(6):e19788. doi: 10.1371/journal.pone.0019788 (PMC3110611; doi:10.1371/journal.pone.0019788)
Supplement: Table S3 — Respiratory outbreaks monitored by the Mountain Gorilla Veterinary Program (MGVP) between 1986 and 2008. (DOC) [file pone.0019788.s010.doc]

**Table S3**. Respiratory outbreaks monitored by the Mountain Gorilla Veterinary Program (MGVP) between 1986 and 2008.

| Year | Group | Group Size | # gorillas with clinical signs | # treated | # Died (treated) | # Died  (not treated) |
| --- | --- | --- | --- | --- | --- | --- |
| 1988 | Group 13 | 13 | 7 | 6 | 2 | 1 |
| 1988 | Peanuts | 7 | 4 | 1 | 0 | 0 |
| 1988 | Group 11 | 10 | 7 | 3 | 0 | 0 |
| 1990 | Susa | 34 | 24 | 4 | 1 | 1 |
| 1992 | Group 5 | 35 | 4 | 1 | 0 | 0 |
| 1996 | Pablo | 35 | 13 | 0 | 0 | 0 |
| 1996 | Shinda | 20 | 13 | 0 | 0 | 0 |
| 2002 | Shinda | 25 | 9+ | 2 | 0 | 1 |
| 2003 | Beetsme | 26 | 9+ | 9 | 1 | 0 |
| 2004 | Susa | 34 | 23+ | 4 | 0 | 0 |
| 2004 | Sabyinyo | 9 | 9 | 2 | 0 | 1 |
| 2006 | Pablo | 63 | 24 | 1 | 0 | 0 |
| 2007 | Kwitonda | 17 | 17 | 0 | 0 | 0 |
| 2008 | Susa | 39 | 39 | 2 | 0 | 0 |
| 2008 | Group 13 | 25 | 24 | 0 | 0 | 0 |
| 2008 | Hirwa | 12 | 11 | 1 | 0 | 0 |
| 2008 | Kwitonda | 17 | 8 | 0 | 0 | 0 |
| TOTAL | | | 245+ | 36 | 4 | 4 |
