# Supplementary material for: Extreme Conservation Leads to Recovery of the Virunga Mountain Gorillas
Source: PLoS One. 2011 Jun 8;6(6):e19788. doi: 10.1371/journal.pone.0019788 (PMC3110611; doi:10.1371/journal.pone.0019788)
Supplement: Text S1 — Supporting Information. (DOC) [file pone.0019788.s011.doc]

Supporting information

a) Study population

b) Leslie matrix models for habituated groups

c) Time-series analyses

d) Additional growth rate calculations

e) Socioecological influences on female reproductive success

f) Ranger based monitoring program

g) Protection and monitoring activities of the DFGFI

h) Veterinary interventions

i) Minimizing the threat of pathogen transmission

j) Uncertainty estimate for the growth rate of unhabituated gorillas

**a. Study population**

Mountain gorillas are found in three national parks of the Virunga Volcanoes (450 km^2^) straddling the boundaries of Uganda (Mgahinga Gorilla National Park), Rwanda (Parc National des Volcans), and the Democratic Republic of Congo (Parc National des Virungas). For 580 of the 668 habituated gorillas (87%), the age is known within two years (Tables S1, S2). The remaining 88 gorillas (13%) were already adults when they were habituated, so their age estimates are less precise (± 4-10 years). Gorillas are classified as immature until they reach age eight, and are considered adults thereafter.

Each of the current habituated groups is generally observed on a daily basis, but before 1980 it was more typical to monitor only one group per day, and all observations have been interrupted at times (e.g., during the civil unrest in 1992-1993, 1997-1998, and in the DRC from 2007-2008). Therefore, the estimated impact of conservation measures may be conservative because they include times when those measures were not in place. For purposes of this study, groups are not considered “habituated” until their membership was fully reported thus some numbers presented here differ slightly from those previous publications [1,2].

**b. Leslie matrix models for habituated groups**

There were 457 births in 1790 adult female-years, which represent an overall birth rate of 0.255 births per adult female-year (Table S2). When each year of the study is considered a separate data point, the birth rates had a standard error of ±0.0116. The birth rate of adult females was not significantly correlated with age (R^2^ = 0.042, F_34,1_ = 1.5, p = 0.23), nor age^2^ (R^2^ = 0.063, F_34,1_ = 2.3, p = 0.14), so the overall value was used for adult females of all ages in all Leslie matrix calculations. Although females are not considered adults until they reach age eight, the database contains three births in 104 female-years at age seven. Therefore we also included a rate of 0.029 births per female-year at age seven in all Leslie matrix calculations. We assumed a 50:50 birth sex ratio [3].

There have been 211 deaths during the 5652 gorilla-years observed, which represents an overall mortality rate of 0.037 deaths per gorilla-year. When each year of the study is considered a separate data point, the mortality rates had a standard error of ±0.00077. In addition to the 211 deaths, there were 51 ‘unexplained disappearances’ with no clear indication if the gorilla had died or dispersed. After controlling for age (Figure S1), survival was not significantly different for immature males versus females when we assumed that the unexplained disappearances were due to death (log rank test: χ^2^ = 0.31, df = 1, p = 0.58) or dispersal (χ^2^ = 0.38, df = 1, p = 0.54). Therefore, to increase sample size and avoid uncertain sex determinations, we included both sexes (and unsexed gorillas) for calculating survival at those ages in the all Leslie matrix models. Survival was significantly lower for adult males than for adult females when we assume that the unexplained disappearances were due to dispersal (χ^2^ = 5.7, df = 1, p = 0.017) but not death (χ^2^ = 3.2, df = 1, p = 0.072). The Leslie matrix models used only females in the survivorship data for adult gorillas.

The Leslie matrix model predicted a growth rate of 2.61% if all unexplained disappearances were due to deaths and 3.65% if they were due to dispersal, which leads to the average value of 3.13% as reported in the main text. Unless otherwise stated, we present only the average value for all other Leslie matrix calculations. As explained in Section C, the range (±0.52%) may represent an upper limit on the uncertainty from unexplained disappearances, because it is highly unlikely that all of the disappearances were entirely due to either deaths or disappearances.

**c. Time-series analyses**

Time series analyses involved iterative calculations using the bisection method in combination with Equations 1 & 2 from the main text [4]. The bisection method is an algorithm which finds the value of “x” that corresponds to a value of zero for a function such as y=f(x). In our case, the parameter for “x” is equivalent to r_m_ in Equation 1. For a given value of r_m_, Equation 1 calculates the number of gorillas at the end of a specified time interval (N_CALC_). The parameter for “y” is equivalent to N_CALC_ minus N_OBS_, in which N_OBS_ equals the observed number of gorillas at the end of the time interval. We ran iterations with the bisection method until the absolute value of “y” was less than a “convergence tolerance” of 0.01. We then used Equation 2 to convert the final value of r_m_ into an annual growth rate.

To examine how our convergence tolerance contributed to uncertainty in the growth rates throughout the study, we ran the bisection method while varying the final population size by ±0.01 gorillas. The corresponding growth rates varied by ±0.000092% for the total population, ±0.000190% for the habituated gorillas, and by ±0.000273% for the unhabituated gorillas. Thus we believe that our convergence tolerance was not an important source of uncertainty in our growth rate estimates.

After adjusting for all exchanges (habituation and dispersal) between the habituated and unhabituated groups, the time-series calculations indicate an average growth rate of 4.11% for the habituated groups. The average value is based upon a growth rate of 3.65% if all unexplained disappearances were due to deaths and 4.57% if they were due to dispersal. The results from time-series calculations can differ from Leslie matrix models due to factors such as demographic stochasticity, and unstable age structure, and covariance among vital rates [5,6]. The time-series calculations indicate a growth rate of -0.37% for the unhabituated groups if unexplained disappearances (from the habituated groups) were due to deaths, and a growth rate of -1.11% if they were due to dispersal, for an average value of -0.74% with a range of uncertainty equaling ±0.37%.

As noted in Section B, the ranges may represent an upper limit on the uncertainty from unexplained disappearances, because it is highly unlikely that all of those disappearances were solely due to either deaths or dispersal. To provide a more conservative estimate of this uncertainty, we repeated the time series analyses 1000 times in combination with a random number generator for values between 0-1. We assumed that an unexplained disappearance was due to death if a random number was less than 0.5, and due to dispersal if the random number was greater than 0.5. We used a new random number for each unexplained disappearance in each calculation of the time series analyses. The standard deviation from 1000 calculations of the time series analyses was ±0.088% for habituated groups and ±0.059% for unhabituated groups for the overall growth rates throughout the study. These standard deviations may represent a lower limit on the uncertainty from unexplained disappearances, because they do not reflect uncertainty about our assumption that those disappearances have an equal probability of being due to deaths versus dispersal. Therefore, the actual uncertainty from unexplained disappearances probably falls between the standard deviations shown in this paragraph and the ranges that are shown in the previous paragraph.

The combined size of all unhabituated groups was taken from data obtained from six censuses of the entire population between 1970 and 2003 (Figure 1a). In addition to the growth rate for the overall study period, we also calculated separate growth rates for the interval before the 1981 census, and the interval since the 1989 census, and the interval in between, to examine temporal variability in the growth rate that may be related to changes in anthropogenic influences (Figure 1). Note that those calculations do not use the results of the 2000 census, which is considered less reliable than the 2003 census due to logistical limitations during the civil unrest [1,2].

The main text noted that habituated gorillas had a higher growth rate than unhabituated gorillas during all five intervals between consecutive censuses, but the paired t-test should be interpreted with caution. Statistical analyses are typically performed on a small sample in order to make inferences about a broader population. In contrast, we have directly measured the entire population of Virunga mountain gorillas, so it does not seem necessary to make broader inferences about their growth rates. The analysis of temporal variability may provide qualitative reassurances that habituated groups will continue to fare better than unhabituated groups, but efforts to improve conservation are continuing, so a statistical analysis of previous data cannot quantify the probability that future results will be similar.

**d. Additional growth rate calculations**

Our reported impacts of veterinary interventions are based on the assumption that each surviving gorilla would have died without treatment (Table 2 in the main text). Although treatment is typically withheld until conditions become life threatening, we ran additional Leslie matrix models which assumed that surviving gorillas would have had a 25%, 50%, or 75% chance of survival without intervention (Figure S2). Those results indicate that any intermediate effectiveness of veterinary interventions can be reliably interpolated between the case studies in the main text (0% survival without treatment), and the base case (which is equivalent to 100% survival without treatment). After accounting for veterinary interventions, the main text claims that increased protection was responsible for most of the remaining difference between the growth rates of the habituated versus unhabituated groups. The rest of this subsection examines other factors that could contribute to those differences.

Even if the habituated and unhabituated gorillas received equal conservation efforts, differences between their growth rates could arise from temporal and spatial variability in the severity of threats that they have faced. For example, this study considers five tourist groups to be “unhabituated” during the early years of military conflict because detailed demographic data was not yet available. If those groups were considered “habituated” instead, we estimate that the overall growth rate would drop from 4.1% to 3.5% for the habituated groups, and it would increase from -0.7% to -0.6% for the unhabituated groups. Those time-series estimates are based on the group sizes reported for the Luwawa, Ndungutse, Rugabo & Rugendo groups in 1990, and the Faida group in 1993 [1]. The estimates assume that those tourist groups had no net dispersal with unhabituated groups while their demographic data was not fully reported. At least 9-14 gorillas were killed in those tourist groups before they were considered habituated (ibid), in addition to the 26 killings reported for all other habituated groups throughout the study. Thus the growth rate of the habituated groups may be inflated because it excludes some groups during years when the human disturbances seemed especially severe.

Even if the habituated and unhabituated groups had the same fertility and mortality rates as a function of age and sex, differences in their short-term growth rates could arise from their age and sex distributions. The habituated groups have contained more immature gorillas, more adult females, and fewer adult males than predicted by a stable age distribution (Figure S3). The skewed distribution arose primarily because dispersing males become solitary rather than joining breeding groups, and because habituation has focused on breeding groups (e.g. most males are not monitored after dispersing). Consequently, the net influx into habituated groups has been 70% female (habituation and immigration minus emigration and end of group monitoring). The time-series analyses and dynamic simulations account for those sex-biased exchanges, but the Leslie matrix models do not. Therefore, if those exchanges had not been sex-biased, then the growth rate of the habituated groups would have been closer to the Leslie matrix model (3.1%) than to the dynamic simulations (3.9%) or the time-series analyses (4.1%). Thus sex-biased exchanges boosted the growth rate of the habituated groups at the expense of the unhabituated groups, but vet interventions and conservation efforts are still largely responsible for the majority of the difference between those two subsets of the population.

**e. Socioecological influences on female reproductive success**

Feeding competition, predation, and infanticide are considered the main socioecological influences upon primates [7-9]. To evaluate whether differences in biomass density or group size could cause spatial or temporal variability in feeding competition and FRS, we considered three dependent variables: the age of first parturition, the length of interbirth intervals with surviving offspring (IBI), and the proportion of offspring that survived to reach age three (Table 1, Figure S4, S5, S6). We also estimated the impact of infanticide upon variability in the population growth rate. The Virunga mountain gorillas currently have no natural predators.

The estimates of biomass density are largely based on a one year time period, but our statistical analyses of FRS included the date as an independent variable to test for temporal variations. The biomass density could have increased after cattle were removed from the park in the 1970s and/or decreased as the gorilla population has grown in recent years. We also tested the date^2^ (a quadratic term) because those two temporal influences may combine to produce nonlinear patterns (i.e., low biomass density in the early and the late years and a maximum biomass density during the intermediate years). In addition, we included an interaction term (group size divided by biomass density) because changes in group size could have more impact on within group scramble competition when the biomass density is low than when food is abundant, and because larger groups may be more sensitive to differences in biomass density than smaller groups [9,10]. None of those additional variables was significant in multivariate analyses so the results are not shown.

Infanticide by mountain gorillas typically occurs when the adult male dies in a one-male group, because the group is taken over by an outsider male [11,12]. By killing the infants, the outsider male ends the lactational amenorrhea of their mothers so he can begin to reproduce more quickly [13]. Infanticide is not expected after the death of a dominant male in multimale groups, because the remaining males may be related to the infants in their group. In the 2003 census, 31 of the 58 (53%) habituated infants were in multimale groups, versus only 1 of 8 (12.5%) unhabituated infants, which could lead to differences in the rates of infanticide and population growth. Nonetheless, when we ran Leslie matrix models based on survivorship data solely from one-male groups, infanticide following the death of a dominant male reduced their predicted growth rate by only 0.2%. Thus we conclude that such infanticide has not been a major source of variability in the population growth rate.

**f. Ranger based monitoring program**

The Ranger Based Monitoring program involves monitoring and data collection conducted by park staff on daily patrols, and use of this information for day-to-day management and conservation [14]. Three rangers generally stay with each habituated tourist group for an average of 4 to 5 hours per day, although this can change depending on the location of the group and the amount of time needed to find them. During the one hour visitation for tourists, the guidelines state that the guides and tourists should maintain a distance of 7m between themselves and the gorillas. Otherwise, rangers stay at a distance of approximately 100m, which is far enough to avoid disturbing the gorillas, yet close enough to respond if any incident occurs.

In addition to direct monitoring of habituated groups, rangers also conduct routine anti-poaching patrols throughout the Virunga Massif. The only areas that remain uncovered by patrols (when security conditions are good) are those that are inaccessible due to the rugged nature of the terrain. Signs of unhabituated gorillas are recorded by ranger patrols when they are encountered. If a nest site is encountered then a count will be done to get an estimate of the size of the group. Patrols are deployed to ensure maximum coverage of the park and/or targeted towards areas where there are high levels of illegal activities. The number of rangers in the park on patrol at any one time depends on the nature of the patrol. For example, overnight patrols require more rangers than daily patrols, and coordinated patrols between two adjacent parks also use more staff.

Rangers use established trails to enter the forest and then will make their own trails if they are targeting a specific part of the park. If signs of a poacher are observed then the rangers follow these signs until they locate the illegal activity. Snares, beehives, and poacher’s camps are destroyed. Although the killing of six gorillas in 2007 was linked to people involved with charcoal production, such illegal deforestation has mainly occurred in the Nyamulagira sector, which does not contain any mountain gorillas. Currently there are no cattle in the park, however such grazing has previously contributed to the degradation of gorilla habitat in the Mikeno sector as recently as the early 2000s. Any cattle found by rangers are herded out of the park, confiscated, and their owners fined. The procedure for arresting people inside the park is to follow the judicial system in the country in which they are arrested. This involves close cooperation between the park authorities, local government authorities and the police.

**g. Protection and monitoring activities of the Dian Fossey Gorilla Fund International**

The Karisoke Research Center (KRC) of the Dian Fossey Gorilla Fund International (DFGFI) was established in 1967 by Dr. Dian Fossey. Dr. Fossey’s pioneering efforts to study and protect the mountain gorilla fostered a new understanding of the nature of gorilla behavior and brought international attention to the plight of the population. Her work was featured in magazines such as National Geographic and immortalized in the movie *Gorillas in the Mist*. Dian Fossey’s name remains synonymous with the population and is often cited as a reason tourists come to see the mountain gorillas (KRC, unpublished data).

KRC has continuously monitored and studied a subset of the Virunga mountain gorilla population since 1967, with the exception of one brief interruption just after the Rwandan genocide. Currently, KRC supports the mountain gorilla protection efforts of the Rwandan park authorities by providing daily protection to the groups habituated for research. As of 2011, the research groups are comprised of ~120 individuals living in nine social groups, although traditionally the number of groups has been two to three. All groups have a designated staff team that provides daily protection and monitoring. These teams are in the forest eight hours a day. In addition, KRC research staff are with the groups for approximately four hours during the middle of the day, collecting behavioral, demographic and other data for long-term records. A maximum of three people are with the gorilla group at anyone time and following the visit of researchers the protection and monitoring staff remain in the general vicinity of the gorilla at a distance of approximately 100m. All DFGFI staff follow established visitation guidelines as outlined below^i^.

In addition to direct, daily protection of the research groups, DFGFI also conducts protection activities in the area of the park where these groups live (often termed the Karisoke sector). A team conducts anti-poaching patrols throughout the Karisoke sector of the park. During the patrols, the presence and location of illegal activities, such as wood or water collection, are recorded and snares are removed (roughly 1000 per annum). All data on snares and other illegal activities are shared with the Rwandan park authorities to help guide park management decisions. Karisoke’s anti-poaching team also works with staff from the Rwandan park authority to patrols areas outside the Karisoke sector of the park. Team members set up campsites from which they jointly patrol more remote areas of the Volcanoes National Park for extended periods of time. Finally, Karisoke staff conduct cross-border patrols with local teams from the Ugandan and Congolese sectors of the gorillas’ habitat.

**h. Veterinary monitoring and interventions**

The Mountain Gorilla Veterinary Project, established in 1986, is one of the few organizations that provides *in situ* veterinary treatment for wild animals [15]. The mission of MGVP is to intervene only in cases of human-induced and life-threatening illness, without causing undue disruption to the gorillas. This includes cases when the injury/illness was likely caused by humans (snares and respiratory disease), when there is the risk that the illness can spread to other individuals (e.g. respiratory disease), or when the life of an individual is at risk and the intent is to minimize mortality to breeding females or silverbacks who are the leaders of single male groups (includes treatment of gorilla-induced wounds and other injuries and illnesses; hereafter considered as ‘other’)**.**

The purpose of the intervention policy is to conserve the maximum genetic variability of the population by saving animals that have the highest potential to do that. Clinical judgment is used during respiratory outbreaks since often there is high morbidity and low mortality. Therefore only animals showing severe clinical signs of extreme moist coughing, inappetance and lethargy for several consecutive days are treated. If it were a pathogen causing acute high mortality in the group then more aggressive therapy would be implemented.

Outbreaks of group-wide respiratory disease have occurred in 10 of the past 22 years among Rwanda’s habituated mountain gorillas, and were characterized by coughing, sneezing, lethargy, and partial anorexia (Table S3) Once respiratory illness appeared in a group of gorillas, the problem was monitored every one to three days by the field vets along with the park ranger or DFGFI staff, depending on the group, and the park veterinary warden. If the ill animal resided in a tourist group, veterinary check visits were performed with the tracking team early in the morning, before the arrival of tourists. If necessary, the veterinarians resumed their monitoring after the tourist visit.

During each veterinary visit, a standard set of observational data was collected for each gorilla. All individuals were identified by their distinctive nose print patterns. All observations were based on a visual exam that lasted from 7 to 30 min. The data recorded included activity level, appetite, attitude, mucous membrane color, respiratory rate, rhythm and quality, overall body condition, abdominal fullness, mobility, urine color, fecal consistency, and any changes associated with the eyes, ears, mouth, skin, hair, or limbs. The following were considered clinical signs of respiratory illness: 1) sneezing; 2) nasal discharge; 3) coughing; 4) dyspnea or tachypnea; 5) evidence of headache, based upon prolonged head-holding or head-tapping; 6) anorexia, lack of foraging; 7) shivering, based upon visible goose bumps on the skin surface; this sign was considered evidence of fever; and 8) weakness, difficulty keeping up with the group. Coughing gorillas that showed all three of these last signs—anorexia, weakness, and shivering—were considered systemically ill and were monitored closely.

About half the time, the disease course was mild and the gorillas recovered within one to two weeks. But in other cases the problem was more severe and lasted for a month or longer. These gorillas developed loud, harsh, spasmodic coughs. Most gorillas improved gradually over the course of several days, but some continued to cough and developed signs consistent with life-threatening pneumonia, including anorexia, weakness, and shivering. Veterinary intervention was performed on severely ill gorillas only after careful consideration of the disease course, and the potential disruption to the gorilla group from the darting. It should be noted that sick gorillas often lag behind the group. Thus the disruption from intervention was often less than when treating an otherwise healthy gorilla with a snare.

We defined an intervention as any time a human provided medical/health care to a gorilla, which is consistent with general medical practice. This includes manual restraint for a snare removal, darting with antibiotics or a vaccine, or a full immobilization. Snare removals performed by field staff (n = 10) are included in the database and analysis. Sometimes multiple interventions were required to treat a single case. However, for this study, we consider incidents requiring multiple interventions as one, because we are interested in measuring the impact of human interventions per illness/snare. These multiple interventions to treat one illness are not independent events.

There were 6 times that adult females were anesthetized so the veterinarians could treat their dependent infants. For our analysis, we are considering these as interventions for the infants (n = 6) only and not the mothers. Cases of anesthetizing a mother to recover a dead infant (n = 4) were not included because they were not performed to provide medical care to the gorilla. Three interventions to treat three gorillas that were agonal (near death) were not included in the database or analysis because no amount of veterinary care could have prevented the death. We were unable to include 14 interventions in the analysis because they occurred in groups before we had reliable demographic data or we were unable to match the names of the gorillas between the demographic and veterinary databases. We also did not analyze the impact of vaccinating 65 of the 103 habituated gorillas (63%) for measles following a respiratory outbreak in 1988 because it is impossible to measure the potential impact this had on the population and because the presumptive diagnosis of measles was based upon necropsy findings from 2 gorillas that died early on in the outbreak but was not confirmed in subsequent deaths or in future outbreaks. Additionally, no gorillas have been found to have a positive measles titer other than those who were vaccinated.

**i. Minimizing the threat of pathogen transmission**

The following visitation guidelines represent the rules relevant to disease transmission currently being followed in most settings where great ape ecotourism takes place in central Africa. They are intended also to be relevant to programs with Grauer’s gorillas, lowland gorillas, chimpanzees, and bonobos. Given that disease transmission is two-way, these rules are designed to protect both people and apes.

The maximum number of people visiting a gorilla group for tourist visits is 8 visitors and 2 park staff, all above the age of 15 years old. Typically less than 4 people visit research groups. Everyone is requested to wash their hands, wear clean clothes, and wash their shoes before entering the forest. Everyone (tourists, researchers, and park staff) are asked to report if they are not feeling well (e.g. diarrhea or a sore throat) and not visit the gorillas if they are sick.

Only one 1-hour visit is made to each gorilla group daily by tourists. Researchers typically spend about four hours with the groups habituated for research. Everyone is expected to maintain a 7-meter (23 feet) distance from the gorillas. Rules prohibit eating, drinking, and smoking and no food is given to the gorillas. Currently tourists visiting mountain gorillas in the Democratic Republic of Congo are requested to wear surgical masks. All staff, researchers, and tourists are recommended to wear an N95 mask when visiting the gorillas. If N95 masks are unobtainable and/or too expensive, a standard surgical mask should be used. Pre-visit vaccinations for tourists should be considered. However, vaccinations would not prevent the diseases of prime concern (influenza, common cold, tuberculosis).

Careful monitoring of the habituated gorillas must occur for early detection and a rapid response to any disease outbreaks. Park staff and researchers visiting the gorillas are trained to observe for any signs of illness or injuries and to report them to senior park management and the veterinarians. During a respiratory disease outbreak, and for one week afterwards, staff should not move between groups to minimize the risk of infecting another gorilla group. Other aspects of an emergency management plan include possibly stopping tourist visits to the infected group.

Despite their lower growth rate throughout this study, it is recommended that some gorillas remain unhabituated as a bet hedging strategy. Whereas habituation enables the veterinarians to intervene in case of disease or snares, it also increases the risk of human pathogens being transmitted to the gorillas. Scenarios where habituation increases the risk of a population-wide disease outbreak include transmission among gorilla groups as well as multiple cases of human to gorilla transmissions. Habituated gorillas could also be more vulnerable if humans transmitted a highly lethal disease that resisted veterinary treatment.

**j. Uncertainty estimate for the growth rate of unhabituated gorillas**

The main text reports an uncertainty of ±0.1% for the growth rate of unhabituated groups throughout the study, which was based on uncertainty in the census measurements for the number of unhabituated gorillas. Census documentation has typically reported the total number of gorillas counted (*T_count_*), as well as an estimated population size (*T_est_*) based on the number of gorillas that might have been missed [e.g., Table 2 in reference 1]. We calculated the corresponding quantities for unhabituated gorillas by subtracting the number of habituated gorillas (H_known_), which is known exactly from daily observations of habituated groups (i.e., *U_count_ = T_count_ – H_known_*, and *U_est_ = T_est_ – H_known_*). The estimated proportion of unhabituated gorillas that got counted (rather than missed) in each census was defined as *P_count_ = U_count_ / U_est_*.

To describe the uncertainty in *P_count_*, we used the negative binomial distribution function in Excel 2003 to estimate the probability that a specified quantity of gorillas were missed. In that function, “*Number_f*” equaled the specified number of gorillas missed (the independent variable in the calculation), “*Number_s*” equaled *U_count_*, and “*Probability_s*” equaled *P_count_*. In the 1972 census, for example, *U_count_* = 232, *P_count_* = 95%, and the negative binomial distribution function showed a 3% probability that exactly seven unhabituated gorillas were missed (Figure S7). Thus we estimated a 3% probability that the Virungas contained exactly 232+7 = 239 unhabituated gorillas. In the 2003 census, *U_count_* = 84, *P_count_* = 80%, and the negative binomial distribution function showed a 1% probability that exactly eleven unhabituated gorillas were missed (for a total of 95 unhabituated gorillas). The product of those two probabilities (*W_239,95_* = 0.03%) was defined as the weighting factor for that particular combination of possible population sizes.

The negative binomial distribution showed only a 0.01% probability that the 1972 census missed more than 30 unhabituated gorillas, so we assumed that the Virungas contained from 232 (*U_count_*) up to 262 (*U_count_* + 30) unhabituated gorillas, which represents a total of 31 possible integer values. Similarly, we assumed that the Virungas contained from 84 up to 128 unhabituated gorillas during the 2003 census, for a total of 45 possible integer values. We used time series analyses to calculate a separate growth rate for each possible combination of those two population sizes (31x45 = 1395 calculations). The growth rates from those 1395 calculations ranged from -1.3% to 0.1%, but the weighting factors for those end points (W_262,84_ and W_232,128_) indicate that they have less than 0.0000001% probability of representing the actual growth rate. The uncertainty estimate of ±0.12% that is presented in the Methods of the main text equals the weighted standard deviation of all 1395 possible growth rates for the unhabituated gorillas, using the weighting factors *W_i,f_* for each combination of initial (*i*) and final (*f*) census population sizes.

**Supplemental references**

1. Kalpers J, Williamson EA, Robbins MM, McNeilage A, Nzamurambaho A, et al. (2003) Gorillas in the crossfire: population dynamics of the Virunga mountain gorillas over the past three decades. Oryx 37: 326-337.

2. Gray M, McNeilage A, Fawcett KT, Robbins MM, Ssebide JB, et al. (2009) Census of the Virunga mountain gorillas: complete sweep method versus monitoring. Journal of African Ecology 48: 588-599.

3. Robbins AM, Robbins MM, Fawcett K (2007) Maternal investment of the Virunga mountain gorillas. Ethology 113: 235-245.

4. Byrne CL (2008) Applied Iterative Methods. Wellesley, Massachusetts: A K Peters, Ltd. 400 p.

5. Morris WF, Doak DF (2004) Buffering of life histories against environmental stochasticity: Accounting for a spurious correlation between the variabilities of vital rates and their contributions to fitness. American Naturalist 163: 579-590.

6. Dunham AE, Akcakaya HR, Bridges TS (2006) Using scalar models for precautionary assessments of threatened species. Conservation Biology 20: 1499-1506.

7. Wrangham RW (1980) An ecological model of female-bonded primate groups. Behaviour 75: 262-300.

8. van Schaik CP (1983) Why Are Diurnal Primates Living in Groups. Behaviour 87: 120-144.

9. Sterck EHM, Watts DP, van Schaik CP (1997) The evolution of female social relationships in nonhuman primates. Behavioral Ecology and Sociobiology 41: 291-309.

10. Isbell LA (1991) Contest and scramble competition: patterns of female aggression and ranging behavior in primates. Behavioral Ecology 2: 143-155.

11. Fossey D (1984) Infanticide in mountain gorillas (*Gorilla gorilla beringei*) with comparative notes on chimpanzees. In: Hausfater G, Hrdy S, editors. Infanticide: comparative and evolutionary perspectives. New York: Aldine, Hawthorne. pp. 217-236.

12. Watts DP (1989) Infanticide in mountain gorillas - new cases and a reconsideration of the evidence. Ethology 81: 1-18.

13. Hrdy SB (1974) Male-male competition and infanticide among the langurs (*Presbytis entellus*) of Abu, Rajasthan. Folia Primatologica 22: 19-58.

14. Gray M, Kalpers J (2005) Ranger based monitoring in the Virunga-Bwindi region of East-Central Africa: A simple data collection tool for park management. Biodiversity and Conservation 14: 2723-2741.

15. Cranfield MR (2008) Mountain Gorilla research: The risk of disease transmission relative to the benefit from the perspective of ecosystem health. American Journal of Primatology 70: 751-754.
